# SPIFFI enables single-shot super-resolution and multidimensional imaging

**DOI:** 10.1038/s41592-026-03196-6

**Published:** 2026-08-31

**Authors:** Wei Guo, Lely Feletti, Aleksandra Radenovic

**Affiliations:** Laboratory of Nanoscale Biology, Institute of Bioengineering (IBI), School of Engineering (STI), École Polytechnique Fédérale de Lausanne (EPFL), Lausanne, Switzerland

**Keywords:** Super-resolution microscopy, Single-molecule biophysics, Fluorescence imaging, Polarization microscopy, Image processing

## Abstract

Fluorescence super-resolution microscopy has advanced optical imaging into the nanoscale regime, transforming biological and interdisciplinary research. However, wide-field super-resolution techniques often compromise temporal resolution, thereby limiting the capture of rapid and transient biological events in living systems. Here we introduce spatial polarization-induced fluorescence fluctuation imaging (SPIFFI), a multichannel polarimetric method for single-shot super-resolution imaging and six-dimensional information extraction. By leveraging the inherently smaller point spread function under polarized detection and capturing polarization-dependent spatial fluctuations across multiplexed channels, SPIFFI achieves instant resolution enhancement from a single exposure. This capability substantially enhances the feasibility of volumetric live-cell super-resolution imaging. Moreover, SPIFFI images can integrate seamlessly with existing fluctuation-based methods for further postprocessing and resolution improvement. We demonstrate the versatility of SPIFFI through experiments on both fixed and live cells, capturing rapid subcellular dynamics and enabling high-throughput, multidimensional imaging beyond the diffraction limit. SPIFFI thus offers a practical and robust platform for real-time super-resolution imaging in biological research.

## Abstract (web summary)

Using a polarimetric approach, SPIFFI enables real-time super-resolution imaging of live-cell samples.

## Main

Super-resolution fluorescence microscopy has revolutionized biological imaging by surpassing the diffraction limit^1–6^, achieving nanometer-scale resolution and now approaching the subnanometer level^7–9^. Although it has yet to reach the structural detail of cryo-electron microscopy, optical super-resolution uniquely enables visualization of subcellular dynamics in live cells, which is a critical advantage for cell biology and biomedical research^10–17^. However, current super-resolution techniques face substantial trade-offs. Single-molecule localization microscopy (SMLM) requires tens of thousands of frames and is generally limited to fixed samples^13^. Stimulated emission depletion microscopy places stringent requirements on dye properties and laser power^18^, resulting in substantial phototoxicity for live-cell imaging. Therefore, techniques offering moderate resolution but faster imaging speeds and lower phototoxicity, such as structured illumination microscopy^19^ (SIM), super-resolution optical fluctuation imaging^14^ (SOFI) and super-resolution radial fluctuations^20^ (SRRF), are more compatible with live-cell imaging.

Although SIM is well-suited for live-cell imaging due to its low phototoxicity and fast acquisition^19^, its resolution is limited to roughly twice the diffraction limit and is often affected by reconstruction artifacts. To mitigate these issues, post-processing approaches such as deconvolution^21–23^ and deep learning^24,25^ have been developed to improve image quality. SOFI, on the other hand, mathematically bypasses the diffraction limit by calculating higher-order temporal cumulants of fluorescence fluctuations, which nonlinearly shrinks the effective point spread function (PSF). However, for weakly fluctuating fluorescence signals or images with low signal-to-noise ratios (SNRs), hundreds of frames are still needed to suppress noise and reduce reconstruction artifacts^13,26,27^, and the number of images required for higher-order SOFI reconstruction may also rise exponentially. Therefore, the application of self-blinking dyes^28^ and a two-step deconvolution method^29^ have been proposed to separately address the above issues that hinder the practicality of SOFI. Although SOFI has recently shown potential to be used in applications comparable to SIM, robust imaging for higher-order SOFI remains a challenge^27^. Current real-time super-resolution methods typically involve reconstruction across multiple frames with sliding temporal windows^19,26,30^, which are sensitive to movement artifacts in live cells. To overcome this temporal limitation, single-shot super-resolution techniques such as instant SIM^31^ and image scanning microscopy^5^ have been developed. Unlike conventional SIM, these methods achieve moderate resolution enhancement primarily through optical hardware design and image reassignment, with minimal postprocessing and fewer reconstruction artifacts. With the development of super-resolution microscopy, researchers have increasingly recognized that multimodal and multidimensional information is crucial for studying complex cellular dynamics. Consequently, additional information such as multispectral signals^32,33^ and molecular orientation^34^ has gradually been incorporated into super-resolution imaging approaches.

In this work, we present spatial polarization-induced fluorescence fluctuation imaging (SPIFFI), a single-shot super-resolution technique that exploits the polarization properties of fluorescent dipoles in their excited states. The spread function of a fluorescent dipole under polarized detection inherently exhibits a smaller waist size than the isotropic PSF typically modeled in conventional fluorescence microscopy. By introducing controlled polarization contrasts across multiple detection channels, SPIFFI uses physically constrained spatial fluctuations that enable an instant resolution enhancement of at least 1.7 times. These single-frame super-resolved images can be further processed through a dual-stage reconstruction strategy, achieving a final resolution of ~80 nm without introducing detectable reconstruction artifacts. Beyond resolution improvement, the intrinsic polarimetric design of SPIFFI also enables direct readout of fluorescent dipole orientation and anisotropy. These added dimensions open powerful opportunities for investigating the spatiotemporal organization and functional anisotropy of biomolecules within their native cellular context.

## Results

### Working principle and verification of SPIFFI

**Fig. 1:**
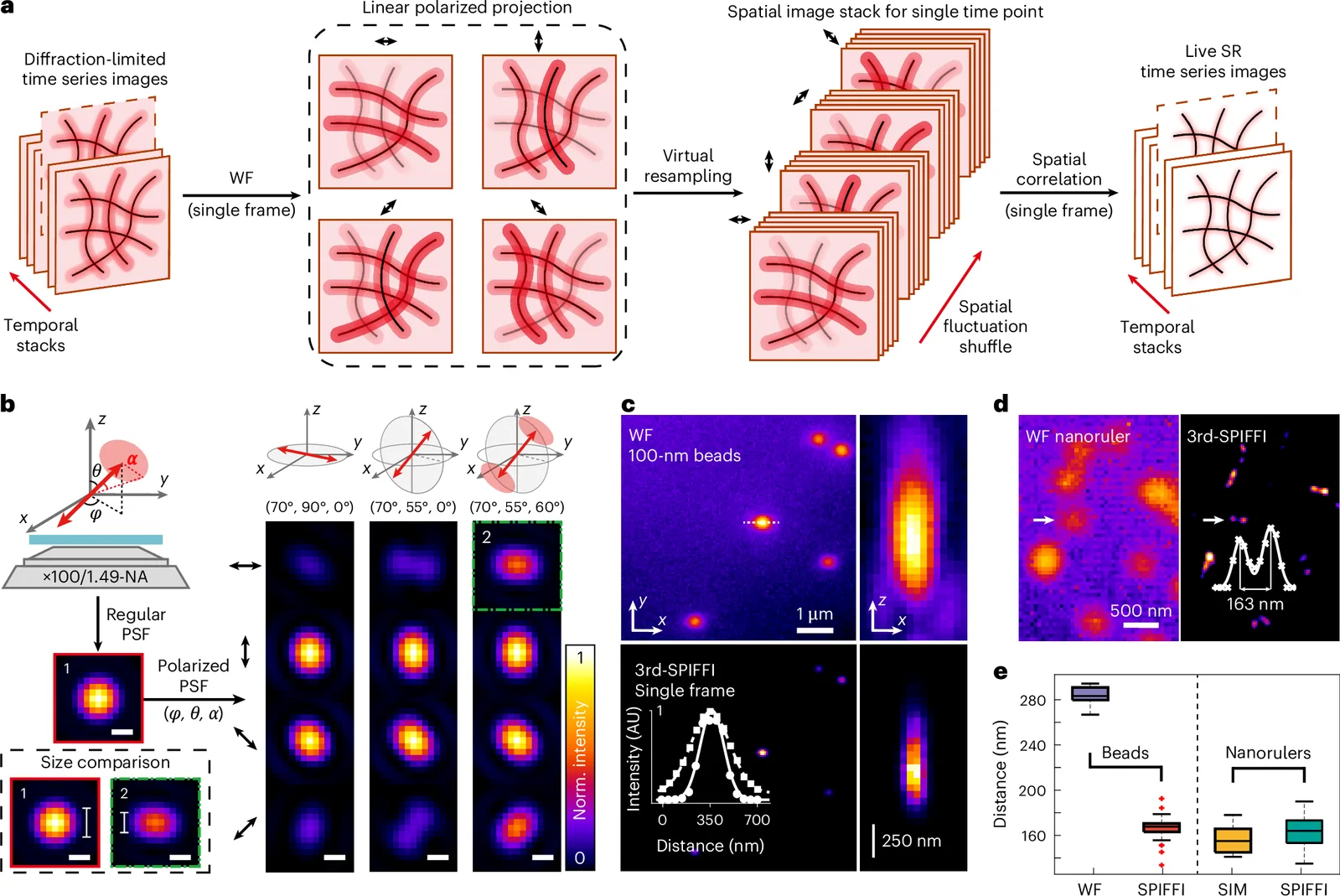
Working principle and resolution validation. **a**, Workflow of SPIFFI based on spatial fluctuations captured across four polarization-resolved channels. **b**, Simulated polarized PSFs of fluorescence dipoles with varying orientations (azimuthal angle *φ* and zenithal angle *θ*) and restricted wobbling (wobble angle *α*). The slimmer waist of each polarized PSF indicates a smaller diffraction limit than the conventional isotropic PSF. All results are simulated under the same conditions and displayed in the same scale. Scale bars, 200 nm. **c**, Lateral and axial cross-sections of 100-nm-diameter beads (emission wavelength of 580 nm). The top image shows a single widefield image, and the bottom image shows the corresponding SPIFFI reconstruction. The inset shows a comparison of the intensity profile of the selected bead. Axial slices were acquired with 50-nm scanning steps. **d**, Widefield image of 160-nm nanorulers (left) and a third-order SPIFFI image (right) reconstructed from a single widefield frame. The intensity profile of a representative nanoruler was selected for size verification. **e**, Resolution evaluation using 100-nm-diameter beads and 160-nm-long nanorulers. The lateral FWHM of the PSFs was analyzed for individual beads, and the structural profile was evaluated for nanorulers to benchmark spatial resolution across different imaging modes. The distance of the nanorulers and PSF size of the beads were obtained by statistically analyzing 20 samples. For the box plots, the center line represents the median, and the bounds of the box indicate the 25th and 75th percentiles. The whiskers extend to the minimum and maximum values within 1.5× the interquartile range, and outliers are plotted individually as points. SR, super-resolution; WF, widefield; Norm., normalized; 3rd-SPIFFI, third-order SPIFFI; AU, arbitrary units. Source data

Because most fluorescent dyes in biological imaging emit from dipolar excited states^35^, we exploit this property by splitting the emission into four detection channels, each detecting a distinct linear polarization state (Supplementary Notes 1–3 and Supplementary Figs. 1–4). As illustrated in Fig. 1a, a single widefield image was decomposed into four polarized subimages, each exhibiting spatial intensity variations due to the angular orientation of the fluorophore’s dipole. However, we shift the fluctuations from the temporal to the spatial domain with the help of multichannel polarimetry, in contrast to conventional SOFI’s reliance on time-resolved fluctuations. To replicate SOFI’s advantages in noise suppression and contrast enhancement, we introduce background subtraction and virtual resampling on four-channel images for data augmentation, which is inspired by denoising models^36–38^ (Supplementary Note 4 and Supplementary Fig. 5). First, background subtraction, based on a rolling-ball algorithm, removes out-of-focus background. Next, because of the symmetrical shape of the polarized PSF, virtual resampling is performed by checkerboard downsampling before recovering the image size using the Delaunay triangulation-based interpolation, creating a noise pair. In parallel, another noise pair from orthogonal-direction interpolation is simultaneously generated due to the directionality of triangulation, increasing the receptive field of spatial neighbors. This step effectively expands the receptive field for neighboring pixels involved in the interpolation process, thereby enhancing the ability to capture subtle spatial fluctuations across polarization channels. Each channel thus produces 5 fluctuation images (1 original + 4 synthetic images), resulting in 20 spatially fluctuating fluorescence images in total. Additionally, we apply predeconvolution and image shuffling to enhance spatial fluctuations, because these two steps can alleviate reconstruction instability caused by uneven intensity ratios across the four polarized channels. Finally, the autocorrelation calculation is used to achieve single-shot super-resolution reconstruction. In this way, a super-resolved temporal image is generated for each frame without relying on time-lapse acquisition or sliding windows, thus avoiding motion artifacts commonly encountered in live-cell imaging. A detailed flowchart can be found in Supplementary Note 5 and Supplementary Figs. 6 and 7.

The resolution enhancement of SPIFFI arises from three aspects: (1) the small size of polarized PSFs, (2) polarization-modulated fluorescence fluctuations^39^ and (3) structural fidelity enhancement benefiting from image resampling. As shown in Fig. 1b, the widefield PSF is usually approximated as a two-dimensional Gaussian shape for an isotropic point source with an emission wavelength of 580 nm, but for a dipole with three degrees of freedom (azimuthal angle *φ*, zenithal angle *θ* and wobble angle *α*), polarized PSFs are mostly elliptical, and their waist size is naturally smaller than that of the widefield PSFs. This direct size reduction is also robust to the orientation and rotational mobility of the fluorescent dipole under the same simulation conditions, which directly ensures that the resolution of the polarized fluorescence image is smaller than the conventional scalar diffraction limit (Supplementary Note 6 and Supplementary Fig. 8). When the dipole wobbles uniformly in a hard cone (that is, restricted wobbling), which is common in fluorescent labeling, its rotational diffusion can also blur the polarized PSF splitting caused by a small zenithal angle and reduce imaging ambiguity. In addition, the second and third points mentioned above usually work in parallel. SPIFFI ensures that the fluctuation analysis is performed directly on the same molecule, which directly shrinks the PSF and avoids image degradation caused by image drift. Moreover, virtual resampling and background subtraction create an approximate zero-mean background signal distribution, so that the correlation of background noise is much smaller than that of the fluorescence signal, resulting in noise suppression and contrast improvement. This level of noise suppression is not achievable by methods such as Richardson–Lucy deconvolution, which iterates to noise-dominated artifacts^40^. Finally, the reduction of the PSF waist size is not isotropic, and it is strongly correlated with dipole orientation, but the autocorrelation calculation is independent of channel selection and fully uses the spatial polarization-induced fluctuations. By simulating two adjacent dipoles with distinct orientations, we illustrate how orientation-dependent polarized PSFs generate significant spatial intensity contrast across different channels. This mechanism enables the resolution of subdiffraction nanoscale structures by introducing spatial fluctuations (Supplementary Note 7 and Supplementary Fig. 9).

We validated SPIFFI’s performance using 100-nm-diameter beads (emission wavelength of 580 nm) and DNA nanorulers. Figure 1c shows three-dimensional fluorescence images of 100-nm-diameter beads with an axial scanning step of 50 nm. The lateral full-width at half-maximum (FWHM) of the third-order SPIFFI for the selected bead was 173 nm, which was about 1.7 times better than that for widefield (294 nm). The axial resolution of SPIFFI was 385 nm, which was 1.8 times better than that for widefield (710 nm). For structural resolution, we imaged DNA nanorulers (GATTA-SIM 160Y), 160 nm (s.d. = 5) in length, labeled with ATTO 565 on both sides (Fig. 1d), providing a precise two-point distance to benchmark the system’s spatial resolving capability. Using SPIFFI, we successfully resolved the nanoruler structures, measuring the length of a selected nanoruler at 163 nm using double Gaussian fitting. By contrast, nanorulers cannot not be resolved by commercial Airyscan, and the SIM reconstructions suffered from artifacts (Supplementary Note 8 and Supplementary Fig. 10). Importantly, SPIFFI reconstructions were obtained from single raw frames, preserving full temporal resolution for the image stack. The lateral FWHM of SPIFFI was 166 nm (s.d. = 12) for beads (Fig. 1e), while the FWHM of widefield imaging was 284 nm (s.d. = 8). The distance resolved by SPIFFI was 163 nm (s.d. = 15) for 20 pairs of nanorulers, and the distance resolved by SIM was 156 nm (s.d. = 11). In addition, because fluorescent beads can be approximated as freely rotating dipoles, the polarization contrast among the four channels was minimal. Nevertheless, SPIFFI still achieved a 1.7-fold resolution improvement in this undesirable scenario. This suggests that for dipoles with restricted rotational mobility, the performance is expected to be at least comparable (Supplementary Notes 9 and 10 and Supplementary Figs. 11–14). These results establish a solid proof-of-concept for SPIFFI, and we next demonstrate its practicality in biological experiments.

### Single-shot super-resolution imaging of fixed cells

**Fig. 2:**
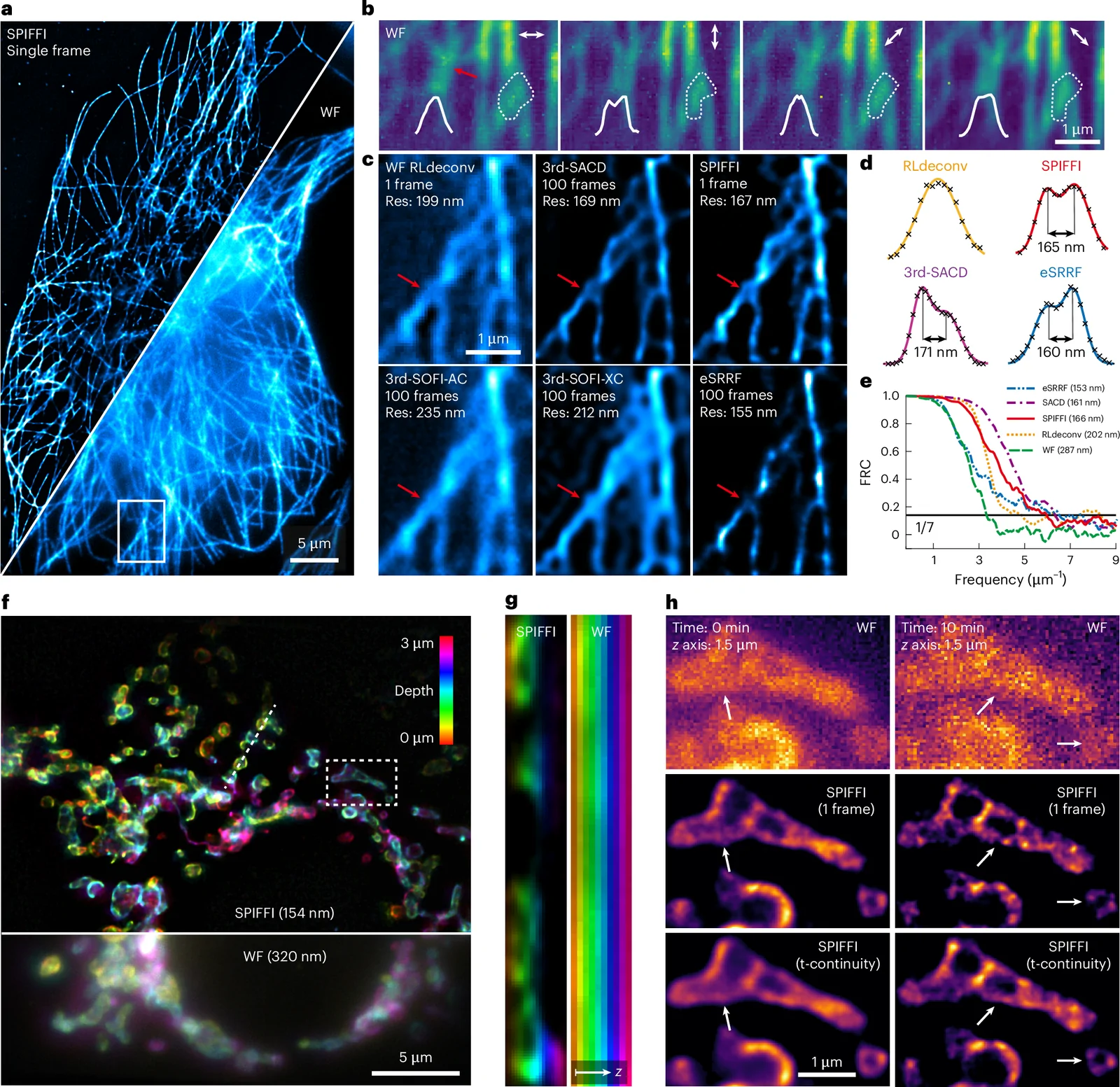
Single-shot super-resolution imaging of fixed cells. **a**, Two-dimensional SPIFFI image of immunolabeled microtubules in a fixed COS-7 cell reconstructed from a single widefield frame, shown alongside the corresponding widefield image. **b**, Averaged widefield images from the four polarization channels. This area was imaged 100 times. The red arrow highlights polarization-dependent intensity fluctuations arising from dipole orientation, and the area encircled by the dotted line shows the contrast difference presented by the polarization measurement. **c**, Performance comparison of several fluctuation-based methods on the same regions. The red arrow marks a fine filament structure that is resolvable in the third-order SPIFFI image. The numbers of frames used in the reconstruction and resolution estimated by decorrelation analysis are marked. **d**, Lateral cross-section and double-Gaussian fitting for the structure marked by red arrows in **c**. **e**, Image resolution determined by FRC analysis using the 1/7 criterion. **f**, Three-dimensional imaging of the mitochondrial outer membrane in an immunolabeled COS-7 cell. Ten axial planes (300-nm steps) are color coded by depth. **g**, Axial cross-section of the dashed line in **f**. **h**, SPIFFI reconstructions of the same region (white box) under high- and low-SNR conditions. Arrows indicate structures whose continuity is improved by a short window. The image fidelity of some arrow-marked structures was improved by temporal continuity (t-continuity). RLdeconv, Richardson–Lucy deconvolution; Res, resolution; 3rd-SACD, third-order SACD; 3rd-SOFI-AC, third-order SOFI-AC; 3rd-SOFI-XC, third-order SOFI with cross-correlation. Source data

We next applied SPIFFI to fixed cells to demonstrate the feasibility of biological imaging. We first performed two-dimensional imaging on immunolabeled microtubules in fixed COS-7 cells (Fig. 2a). When widefield images of each channel are averaged over 100 frames (Fig. 2b), the benefit of polarization measurements can be visualized easily. The intensity profile across two close filaments is plotted as a solid curve, and the contrast of the other structure is highlighted by a dashed box. At the same time, we also display the performance of several fluctuation-based super-resolution techniques (super-resolution imaging based on autocorrelation with two-step deconvolution (SACD)^29^, SOFI with autocorrelation (SOFI-AC)^6^, enhanced SRRF (eSRRF)^30^ and SOFI with cross-correlation^41^) and a widefield image after Richardson–Lucy deconvolution on the same data set (Fig. 2c). Among these methods, both SACD and eSRRF produced a super-resolved image by only using 100 frames. However, benefiting from polarization measurements, SPIFFI showed similar resolution even with single-shot imaging. By contrast, eSRRF suffered from some structural imbalances because of the nonlinearity of fluorescent fluctuation and limited SNR of raw data. SPIFFI enables instant and real-time super-resolution imaging without using any sliding window, which is at least two orders of magnitude faster than the other techniques for showing temporal dynamics. To assess spatial resolution quantitatively, we fitted intensity profiles between two close filaments (red arrows) using a double Gaussian model. SPIFFI resolved a 165-nm structure with a fitting *R*^2^ of 0.9959 (Fig. 2d), while Richardson–Lucy deconvolution failed to resolve it. Additionally, as a benchmark, eSRRF resolved a similar structure (160 nm), and the same structure in SACD was around 171 nm, though its contrast was slightly weaker than that of both eSRRF and SPIFFI. Fourier ring correlation^42^ (FRC) analysis further confirmed SPIFFI’s superior resolution (Fig. 2e): 166 nm by SPIFFI (1 frame), 153 nm by eSRRF (100 frames) and 161 nm by SACD (100 frames). Because both SPIFFI and SACD use the same and best iterations of deconvolution for fair comparison, it can be seen that polarization measurements contribute to more effective extraction of the target’s fine structure. Compared to pure Richardson–Lucy deconvolution using the same number of iterations, SPIFFI still maintained better structural resolution. SPIFFI also exhibited good reconstruction fidelity^43^ compared to other fluctuation-based techniques (Supplementary Notes 11 and 12 and Supplementary Figs. 15 and 16). Moreover, these FRC results aligned with the resolution calculated by decorrelation analysis^18^ in Fig. 2c, which is used for resolution assessment in the following sections because it can directly estimate the resolution of a single image instead of an image pair (Supplementary Note 13 and Supplementary Fig. 17). In addition, we compared SPIFFI with commercial SIM on immunolabeled microtubules (Supplementary Note 14 and Supplementary Fig. 18). COS-7 cells were cultured on gold-marked coverslips and mounted with mounting medium (Fluoromount-G) to enable multi-instrument imaging. SPIFFI achieved comparable structural detail to SIM using a single frame and resolved a 131-nm filament separation.

To demonstrate the advantages of SPIFFI in imaging speed and resolution enhancement, we extended its application to volumetric imaging of the outer mitochondrial membrane in fixed COS-7 cells (Fig. 2f). Using single-shot super-resolution imaging, the intracellular mitochondrial network was visualized by ten axial scanning images with 300-nm axial steps and in color-coded depth. The improved three-dimensional resolution helped us to successfully resolve the hollow structure and intertwined mitochondrial network in both lateral (Fig. 2f) and axial (Fig. 2g) planes, whereas widefield images appeared severely blurred. To improve image fidelity for low-SNR images during longer-term imaging, we also implemented a minimal sliding window strategy to fully use the temporal continuity of fluorescence images. As shown in Fig. 2h, single-shot SPIFFI resolved the outer mitochondrial membrane, but using a sliding window of three frames (plus or minus one frame) could further enhance the integrity and smoothness of the outer membrane structure, as shown by the white arrows. At the 10-min timepoint, the membrane appeared discontinuous in the single-frame SPIFFI image but was restored with the short window strategy. SPIFFI acquired additional polarization-dimension features without losing photons, making the utilization of photons more efficient in the super-resolution reconstruction.

### Dual-stage reconstruction for further resolution improvement

**Fig. 3:**
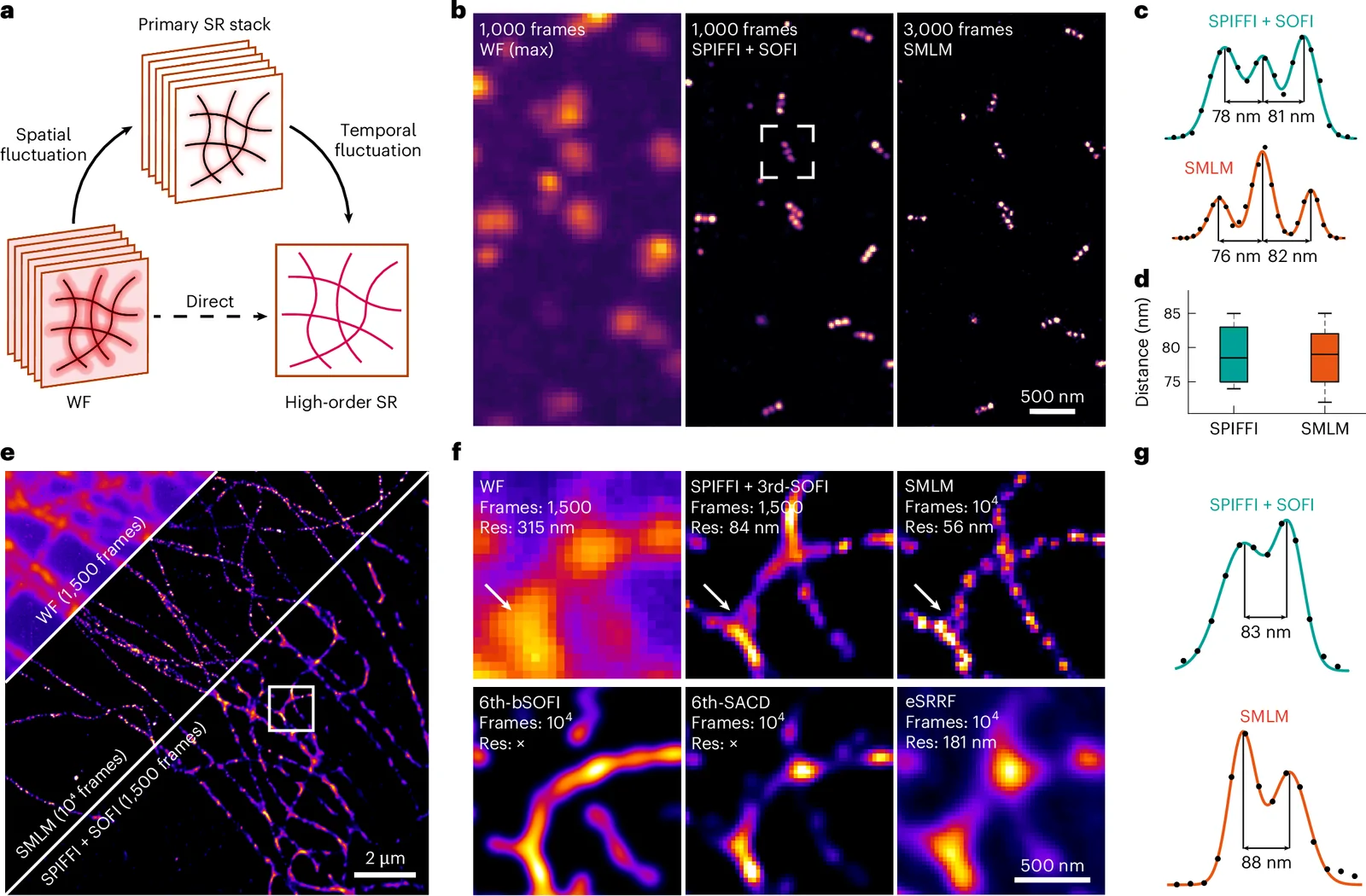
Dual-stage reconstruction achieves 80-nm resolution. **a**, Workflow for the first (spatial fluctuation) and second (temporal fluctuation) reconstructions. **b**, Comparison of widefield, dual-stage reconstruction and SMLM images of DNA nanorulers with 80-nm marker separation. The widefield image is the maximum projection of 1,000 frames; both the two-stage construction and SMLM resolve all three labeled rulers. **c**, Intensity profile of a representative nanoruler demonstrated by simultaneous fitting with three Gaussian functions to evaluate the 80-nm-spaced structural features. **d**, Resolution evaluation using 80-nm-spaced nanorulers. Spatial resolution is benchmarked by evaluating the intermarker distances reconstructed via SMLM and two-stage SPIFFI, respectively. The result was obtained by analyzing 20 pairs of spacings. For the box plots, the center line represents the median, and the bounds of the box indicate the 25th and 75th percentiles. The whiskers extend to the minimum and maximum values within 1.5× the interquartile range, and outliers are plotted individually as points. **e**, Validation of the dual-stage reconstruction of fixed COS-7 microtubules immunolabeled with CF568. **f**, Zoomed-in views of a selected area from **e** and comparison of different methods. **g**, Intensity profile of a selected structure in **f**. 6th-bSOFI, sixth-order balanced SOFI; 6th-SACD, sixth-order SACD. Source data

We further improved resolution by performing a secondary reconstruction on primary super-resolution images, combining both spatial and temporal fluctuations (Fig. 3a). All samples were imaged under blinking conditions because this allows us to use SMLM data from the same samples as a benchmark. Each frame was first processed with third-order SPIFFI to obtain a primary super-resolution image, after which third-order SOFI-AC was applied for a secondary reconstruction. For comparison, SMLM results were constructed using ThunderSTORM^42^. This dual-stage approach robustly resolved 80-nm DNA nanorulers (GATTA-PAINT HiRes 80 G with Cy3B) bearing 3 markers (Fig. 3b) using only 1,000 frames. However, the resolution of the widefield image was only 254 nm (Fig. 3b). Intensity profiles of the same ruler in the dual-stage result and in SMLM yielded similar intermarker distances when fitted simultaneously with three Gaussian functions (Fig. 3c). The distances measured by SMLM and the dual-stage reconstruction were 77.9 nm (s.d. = 4.1) and 78.5 nm (s.d. = 4.0), respectively, which were in the range of the manufacturer’s specification (80 nm (s.d. = 5); Fig. 3d). The dual-stage reconstruction achieved the equivalent of ninth-order SOFI while requiring only 1,000 frames, which is the number of frames normally used for second-order SOFI^13^. Moreover, it avoids the resolution degradation and cusp artifacts of high-order SOFI because of separate low-order calculations from two different fluctuation sources and the direct PSF shrinkage of the polarimetric imaging (Supplementary Note 15 and Supplementary Figs. 19 and 20). However, when higher-order temporal reconstruction is pursued to approach single-molecule localization resolution, dipole-induced PSF asymmetry should be explicitly considered, as significant positional shifts may arise under extreme orientational constraints^44^.

We then validated the 80-nm resolution by imaging immunolabeled microtubules in a fixed COS-7 cell (Fig. 3e). The blinking and reversible switching of CF568 dye was induced by a STORM buffer^45^. Dual-stage reconstruction of 1,500 frames reproduced details comparable to SMLM, but other fluctuation-based methods require more than 10,000 frames to resolve the same structure (Fig. 3f). Double-Gaussian fitting produced an 83-nm spacing between two microtubules (Fig. 3g), close to the 88 nm measured by SMLM. In addition, we emphasize the impact of image drift on long-sequence reconstructions because a lateral drift of approximately 50 nm over 1,000 frames (with an exposure time of 50 ms per frame in our case) is common in experiments. Therefore, we used a cross-correlation correction algorithm^46^ to compensate for drift of the piezo stage. Considering the worst-case scenario under Nyquist sampling, the achievable resolution (that is, <100 nm in our case) cannot exceed twice the drift distance. This prevents reliable recovery of 80-nm nanorulers and the fine microtubule structure without drift correction (Supplementary Note 16 and Supplementary Fig. 21), even though the drift is smaller than the structures that we resolved.

### SPIFFI reveals the fast dynamics in live cells

**Fig. 4:**
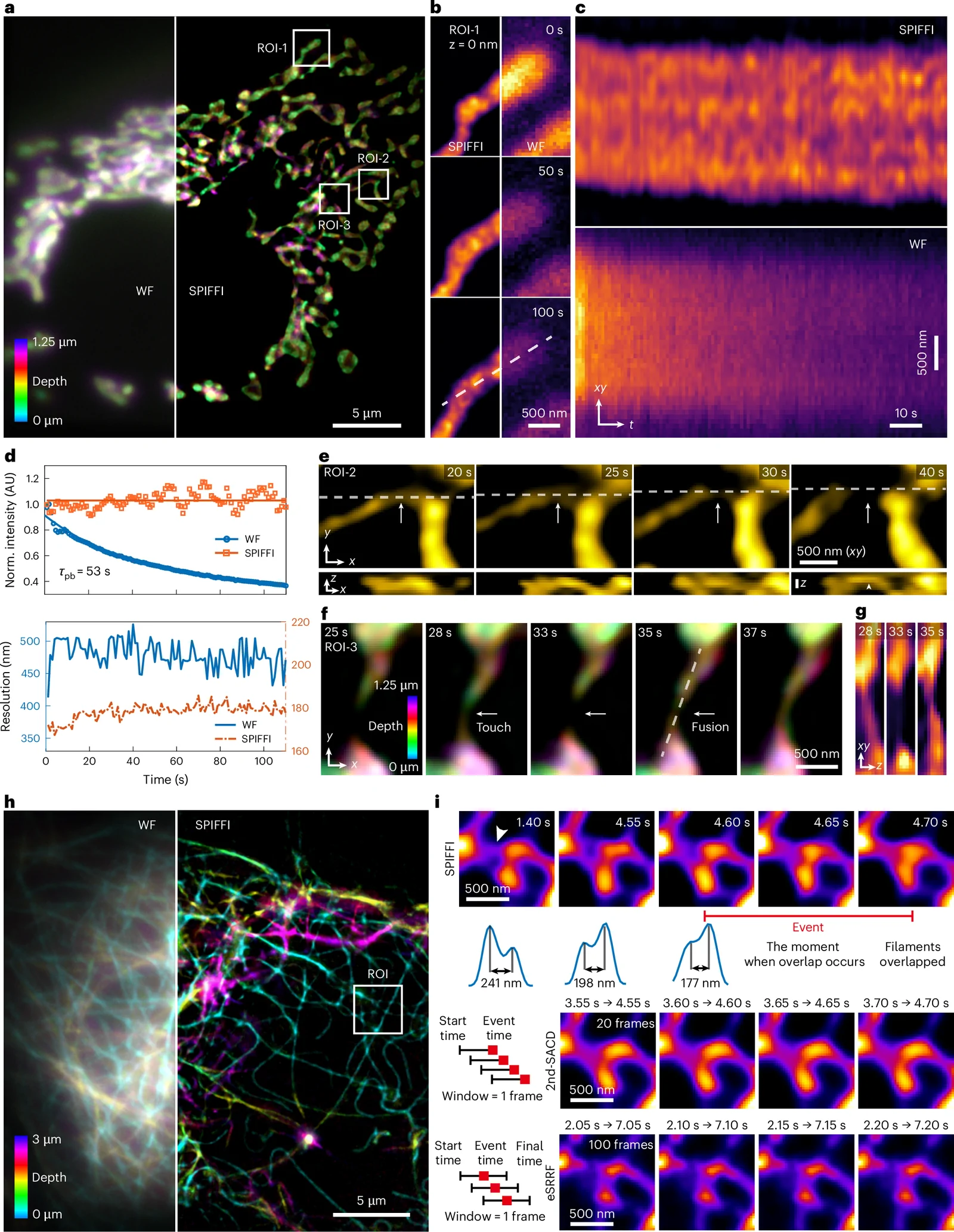
Volumetric super-resolution imaging of live cells. **a**, MIP of mitochondria in a live COS-7 cell labeled with MitoTracker Red. Axial depth of the cell is color coded. **b**, Comparison of SPIFFI and widefield imaging at successive time points for ROI-1. **c**, Space–time plots along the dashed line in **b**. **d**, Reconstruction quality quantification of SPIFFI from image intensity (top) and resolution (bottom). **e**, Selected frames from time-lapse volumetric imaging of ROI-2. Scale bar, 500 nm (*z* axis). **f**, MIP of ROI-3 showing dynamic mitochondrial fusion in four-dimensional (*xyz*-*t*) imaging. The axial depth of the structure is color coded. **g**, Axial profiles of the ROI-3 structure in **f** at 28, 33 and 35 s. **h**, Three-dimensional MIP of microtubules in a live COS-7 cell. The axial depth is color coded using the same color map as in **a** and **f**. **i**, Time-lapse imaging of the selected ROI in **h**. A sliding window with a width of one frame was used to test the performance of the fluctuation-based super-resolution technology on the same data. 2nd-SACD, second-order SACD. Source data

SPIFFI enables fast volumetric scanning, thereby reducing motion artifacts from repeated exposures at the same depth in living cells. As a demonstration, we imaged mitochondria in a live COS-7 cell labeled with MitoTracker Red to highlight the advantages of fast three-dimensional super-resolution imaging for resolving organelle interactions. Images were acquired at five axial planes and rendered as a depth-coded maximum-intensity projection (MIP) in Fig. 4a. SPIFFI maintained high image quality over time in the first slowly moving region of interest (ROI-1; Fig. 4b) and proved resistant to photobleaching (Fig. 4c,d). SPIFFI also resolved the mitochondrial cristae that are not visible under widefield imaging. Even when MitoTracker photobleached with a time constant (τ_pb_) of 53 s, SPIFFI preserved a resolution of 178.4 nm (s.d. = 3.4), whereas the corresponding widefield resolution broadened to 479.0 nm (s.d. = 20.7). In addition, mitochondrial dynamics (for example, fission and fusion) are essential for cellular functional homeostasis^12,47,48^. SPIFFI resolved axial movements with 385.5-nm resolution, showing that a local axial shift at the 30-s mark in ROI-2 could be misinterpreted as fission in two-dimensional imaging alone (Fig. 4e). Similarly, mitochondrial fusion also occurs in three-dimensional space. A tentative axial contact at different depths was observed in ROI-3 at 28 s (Fig. 4f,g). Such prefusion interactions serve as a quality control step, ensuring fusion with only healthy and compatible partners. After the fusion event at 35 s, the newly formed mitochondrion shifted axially to the same imaging depth. This indicates that two-dimensional imaging alone can miss transient prefusion interactions occurring outside the focal plane and may misidentify the actual fusion timing. Similar rapid dynamics can also occur in other cellular structures, such as microtubules. Using fluorescent probes (Abberior LIVE 550) to label microtubules in live COS-7 cells, we collected ten axial planes with 300-nm steps (Fig. 4h). The MIP image revealed the microtubule network spanning the cell, while SPIFFI revealed two microtubules overlapping between 4.6 and 4.7 s. We also demonstrated second-order SACD reconstructed from 20 frames and eSRRF from 100 frames (Fig. 4i). A one-frame-wide sliding window was applied to these reconstructions to capture this rapid dynamic. However, the eSRRF and SACD reconstructions, which must integrate signals over a time-lapse image, were corrupted by motion blur. They failed to resolve the fast dynamics of the microtubules and instead produced blurry, time-averaged results. SPIFFI improves reconstruction efficiency by leveraging the minimized PSF size and polarization contrast, with all images reconstructed directly from high-density widefield data rather than fluorescence blinking analysis.

### Molecular orientations and dynamics in cells

**Fig. 5:**
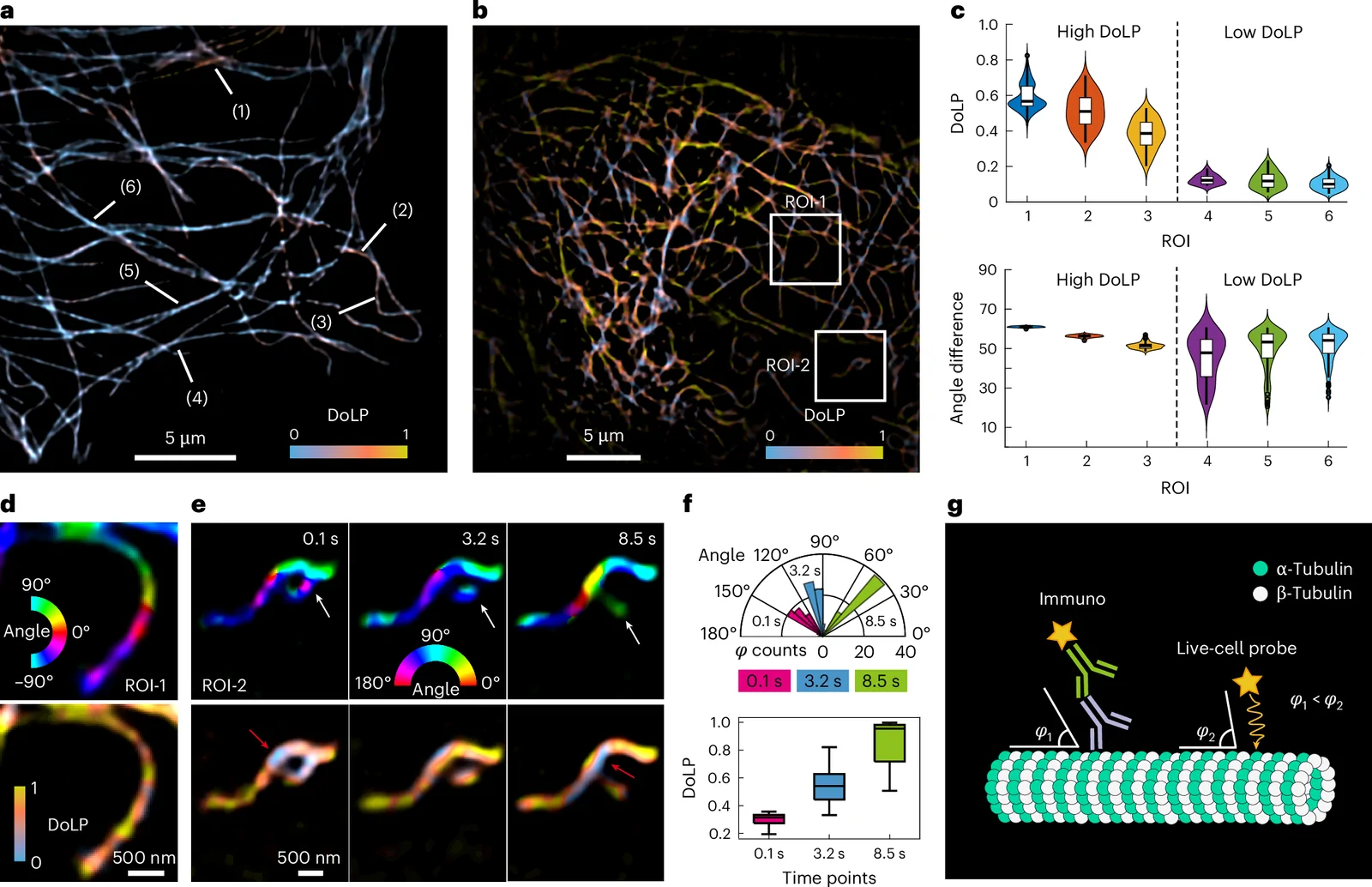
Dipole orientation and dynamics of microtubules with different labeling methods. **a**, DoLP map of immunofluorescence staining. **b**, DoLP map for small-molecule probes used in live-cell imaging. **c**, DoLP distribution and angle difference between molecular dipoles and microtubule directions in the six regions selected in **a**. The numbers of pixels used for the six statistical results are 215, 113, 150, 47, 93 and 120. **d**, Fluorescent dipole information for ROI-1 in **b**. Top: orientation map of fluorescent dipoles. Bottom: DoLP. **e**, Time-lapse fluorescent dipole imaging of microtubules in ROI-2. White arrows in the orientation maps mark the dynamic structure of microtubules, and red arrows in the DoLP images mark the anisotropy of short probes for live-cell imaging. **f**, Angular distribution and DoLP boxplots for the dynamic structures highlighted by white arrows in **e**, quantifying orientation and anisotropy of fluorescent probes over time. The statistical results are from 56 pixels for each ROI. **g**, Comparison of the angle differences between molecules and microtubule directions in the two labeling methods (immunolabeling and live-cell probe). This diagram is only used to illustrate the difference in dipole orientations but does not represent the actual molecular size. For the box plots in **c** and **f**, the center line represents the median, and the bounds of the box indicate the 25th and 75th percentiles. The whiskers extend to the minimum and maximum values within 1.5× the interquartile range, and outliers are plotted individually as points. Source data

SPIFFI can not only achieve fast four-dimensional (*xyz-t*) super-resolution imaging of living cells, but also reveal the orientation and anisotropy of fluorescent dipoles, offering critical insights into the molecular organization of biomolecules in the native cellular environment. For example, antibody length weakens the rigid linkages of molecules in immunofluorescence staining, and the degree of linear polarization (DoLP) map shows that most areas are occupied by strong isotropic signals (that is, low DoLP values; Fig. 5a). By contrast, conjugation of the rhodamine dyes to tubulin-targeting ligands via short linkers led to the formation of rigid complexes with minimal wobble angle, ensuring strong fluorescence polarization after binding (Fig. 5b). We then selected six ROIs (Fig. 5a) to demonstrate the impact of DoLP on dipole-orientation extraction (Fig. 5c). ROI-1–ROI-3 represented somewhat high-DoLP regions, where dipole orientation was relatively fixed. The dipole orientation difference between the immunolabeled CF568 dye and microtubule filament direction was 61.0° (s.d. = 0.4), 56.3° (s.d. = 0.7) and 51.5° (s.d. = 1.6), respectively, corresponding to DoLP values of 0.59 (s.d. = 0.08), 0.51 (s.d. = 0.10) and 0.38 (s.d. = 0.08). By contrast, ROI-4–ROI-6 represented low-DoLP regions (0.13 (s.d. = 0.03), 0.12 (s.d. = 0.05) and 0.11 (s.d. = 0.04)), where dipole angles were 45.7° (s.d. = 11.3), 49.5° (s.d. = 10.7) and 51.5° (s.d. = 8.0), which can be a combined effect of tilted dipoles, dense molecular packing and molecular wobbling. However, benefiting from the ordered orientation of fluorophores on the microtubule in Fig. 5b, the polarized channels captured fluorescence fluctuations with higher sensitivity, enhancing both resolution and angular estimation accuracy. Therefore, in the top image of Fig. 5d, the orientation of fluorescent dipoles in ROI-1 follows the curvature of the microtubule, remaining nearly perpendicular to filaments^49,50^. The DoLP map in the bottom highlights the strong anisotropy of fluorescent dyes in the same region. Meanwhile, in the structure indicated by the white arrows in ROI-2 (Fig. 5e), the orientation of fluorescent dipoles changed as the microtubule filament transitioned from a curved to a relative relaxed state, with measured angles of 136.6° (s.d. = 8.9), 98.4° (s.d. = 5.5) and 48.5° (s.d. = 5.4) at the respective stages in Fig. 5f. The corresponding DoLP increased accordingly, measured at 0.30 (s.d. = 0.04), 0.54 (s.d. = 0.12) and 0.85 (s.d. = 0.16), suggesting that the fluorescent dyes were tightly packed when the filament was curved, thereby reducing the anisotropy of the fluorescence signal. For dyes rigidly linked to targets, a decrease in DoLP typically occurs at filament nodes or bending regions, as exemplified by the structure marked with red arrows in the DoLP map of Fig. 5e. Comparing the two different labeling methods for cell microtubules in Fig. 5g, we found that the projection of the in-plane angle (~83.3°) between the live-cell probe and the filament was slightly larger than that of immunofluorescence (~52.6°). Supplementary experiments with FM4-64 in a giant unilamellar vesicle (GUV) and phalloidin–CF568 on actin filaments further verified SPIFFI’s dipole readout (Supplementary Note 17 and Supplementary Fig. 22). FM4-64 inserts perpendicularly into the membrane on average, restricting wobble and producing relatively high DoLP, whereas dye aggregation on bundled actin reduces DoLP. By performing simultaneous multidimensional imaging of various biological samples, we demonstrate broad potential for real-time studies of subcellular interactions, offering a powerful tool to probe dynamic molecular organization and functional anisotropy in live cells.

## Discussion

The resolution limit in optical fluorescence microscopy is defined by the FWHM of the PSF, below which spatial details cannot be resolved. However, when fluorophores are modeled as fluorescent dipoles, polarization measurements reveal that the waist of their spread functions inherently has a smaller FWHM size, providing a physical foundation for SPIFFI. SPIFFI achieves single-shot super-resolution imaging of live cells by directly reducing the PSF size through autocorrelation of spatially polarized fluctuations, enhancing SNR via background subtraction and improving image fidelity with virtual resampling. Multidimensional dipole imaging simultaneously reveals the native molecular organization and anisotropy within cells. Furthermore, by integrating spatial and temporal fluctuations, SPIFFI achieves 80-nm resolution in fixed samples using only a number of frames comparable to conventional low-order fluctuation-based methods, while effectively suppressing reconstruction artifacts.

Although SPIFFI has demonstrated excellent temporal–spatial–angular performance, several aspects still hold potential for further improvement. For instance, restricted fluorophore rotation (such as in immunofluorescence) and random labeling (such as with MitoTracker) will affect the retrieval accuracy of dipole information. For optimal performance, we suggest using fluorescent probes with short, rigid linkers. Such probes minimize the rotational averaging of the fluorophore’s emission dipole, thereby preserving the intrinsic fluorescence anisotropy. This leads to a higher and more informative DoLP in the widefield image, which enhances the contrast between the SPIFFI channels and improves the accuracy of the orientation measurement. Probes such as fluorescent proteins^49,51,52^ or phalloidin–dye conjugates^53,54^, which offer relatively rigid labeling, are therefore excellent candidates. In addition, labeling density influences the quality of image reconstruction. Sparse labeling would lead to fragmented structures in super-resolved images, which is a general concern that affects the quality of all fluorescence imaging techniques. Membrane-potential-based probes (for example, MitoTracker) label both the inner and outer mitochondrial membranes simultaneously, which can also introduce cusp artifacts and compromise dipole orientation estimation under low-SNR conditions. Therefore, to ensure reliable dipole reconstruction, it is advisable to label distinct structures with spectrally separated fluorescent probes. SPIFFI is inherently compatible with multicolor biological imaging, as its hardware modifications are entirely within the emission optical path (Supplementary Note 18 and Supplementary Fig. 23). Nevertheless, simultaneous multicolor measurement remains an exciting avenue for characterizing organelle interaction^32,33^. Finally, the three-dimensional imaging in this work is still performed by axial scanning with a piezo stage, so integration with direct volumetric imaging techniques, such as multiplane methods^55–57^, could significantly accelerate imaging speed and improve axial resolution.

In summary, we provide new insights into multichannel polarimetry, a configuration already widely used or easily implemented in many labs^53,54,58–60^ in the field of fluorescence anisotropy imaging. Through experiments on various samples (mitochondria, microtubules, actin filaments, nanorulers and lipid membranes) and multiple-resolution imaging modes (single-shot super-resolution, six-dimensional information and dual-stage reconstruction), we demonstrate the flexibility and broad potential of SPIFFI for biological applications, especially for real-time super-resolution imaging.

## Methods

### Imaging setup

The custom-built polarization microscope was configured as follows: the laser (561 nm or 488 nm) was converted into circularly polarized light with more than 95% ellipticity through a combination of a linear polarizer (LPVISE100-A, Thorlabs) and a quarter-wave plate (WPQ05M-561, Thorlabs). To uniformly excite molecules oriented in all in-plane directions, the microscope uses circularly polarized incident light in epi-illumination mode. The sample was mounted on a sample holder attached to a nanopositioning system (Mad City Labs) to ensure precise position control. Fluorescence emitted from the sample was collected using a ×100/1.49-NA oil-immersion objective lens (UPLAPO100X, Olympus), which was optimized for polarization-sensitive imaging. Excitation light was reflected by a dichroic mirror (Di03-R561-t1, Semrock or ZT405-415/488/561/640-phaseR, Chroma), and the emitted fluorescence was filtered using a combination of a 750-nm low-pass filter and 561-nm notch filter to suppress residual excitation light. A variable waveplate (LCC1223-A, Thorlabs) was used in the emission path to correct polarization distortions introduced by the birefringence and differential transmission of the dichroic mirror coating. The fluorescence signal was divided into two optical paths by a nonpolarizing 50:50 beam splitter (BS016, Thorlabs). In one path after the beam splitter, a half-wave plate (AHWP10M-580, Thorlabs) rotated the polarization state by 45°. Subsequently, both optical paths were further separated by polarizing beam splitters (PBS251, Thorlabs), yielding four distinct polarization channels in total. All four channels were finally directed via mirrors onto the same scientific CMOS camera (Prime-95B, Photometrics) for image acquisition.

Airyscan imaging was performed on a Zeiss LSM 980 platform equipped with a ×63/1.40-NA oil-immersion objective and an Axiocam 506 mono detector. For SIM, two commercial systems were used: a Nikon N-SIM system configured with an Apo TIRF ×100/1.49-NA objective and a Photometrics Prime 95B camera and a CrestOptics SIM system equipped with an SR HP Apo TIRF ×100/1.49-NA objective and a Teledyne Kinetix camera.

### Image processing, analysis and visualization

Super-resolution image reconstruction, data analysis and MIP visualization were all based on custom scripts in MATLAB. The widefield images presented in this work were reconstructed ‘pseudo-widefield’ images. Because the fluorescence emission is split into four polarization-resolved subimages, the total intensity image was recovered by the summation of these four channels to provide a diffraction-limited reference for comparison. Visualization of SPIFFI results follows the linearization of bSOFI, but widefield images were left unprocessed. The three-dimensional rendering in Supplementary Videos 2 and 4 was based on the functions of Fiji^61^. The following colormaps were used for three-dimensional rendering: ‘hsv’ (Fig. 2f), ‘biop-12colors’ (Fig. 4a,f,h) and ‘isolum’^62^ (Fig. 4i,j, DoLP). We visualized Fig. 5c using violin and raincloud plots implemented in the MATLAB daviolinplot^63^ toolbox.

### Cell culture

COS-7 cells (605470, Cytion) were cultured at 37 °C with 5% CO_2_ using DMEM without phenol red (21063045, Thermo Fisher Scientific), supplemented with 10% fetal bovine serum, 1% penicillin–streptomycin and 4 mM L-glutamine (A5669701, 15140122 and 25030081, Thermo Fisher Scientific). Cells were seeded at a density of 5,000 cells per cm^2^ on plasma-cleaned, fibronectin-coated 35-mm glass coverslips.

### Fixed-cell labeling

#### Microtubule and mitochondrial labeling

COS-7 cells were prefixed in a prewarmed buffer with 0.25% (wt/vol) glutaraldehyde and 0.3% (vol/vol) Triton X-100. After fixation with 4% paraformaldehyde in PBS buffer, cells were permeabilized with 0.25% (vol/vol) Triton X-100 in PBS and blocked with buffer (2% (wt/vol) BSA, 0.2% gelatin, 10 mM glycine and 50 mM ammonium chloride in PBS; pH 7.4) for 1 h. For microtubule experiments, cells were incubated overnight with monoclonal anti-α-tubulin (T5168, Sigma, 1:1,000 dilution) at 4 °C and washed three times for 5 min each with blocking buffer. Cells were incubated with goat anti-mouse IgG (H + L) CF568 (SAB4600083, Sigma-Aldrich, 1:1,000 dilution) or goat anti-mouse IgG (H + L) Alexa Fluor 488 (A28175, Thermo Fisher) for 1 h at room temperature. For mitochondrial experiments, cells were incubated with rabbit anti-TOMM20 (ab186734, Abcam; 1:1,000 dilution) overnight at 4 °C and then incubated with goat anti-rabbit IgG AlexaFluor 568 (ab175471, Abcam; 1:1,000 dilution) for 1 h at room temperature. The samples were then washed three times for 5 min each with blocking buffer.

#### Actin labeling

Cells were washed three times in PBS and fixed on ice with 4% paraformaldehyde in PBS for 15 min. After washing three more times in PBS, the cells were permeabilized with 0.5% Triton X-100 in PBS at room temperature for 10 min, followed by three washes with PBS. The staining solution was prepared by diluting the stock solution (200 U ml^−^^1^) 1:40 in PBS. Cells were incubated with phalloidin–CF568 (00044, Biotium) for 20 min and washed three times with PBS.

### Live-cell labeling

MitoTracker Red CMXRos (M7512, Invitrogen) was diluted to a working concentration of 50 nM or 100 nM in DMEM supplemented with 5% fetal bovine serum and 1% penicillin–streptomycin. The cell culture medium was removed from the dishes, and a prewarmed staining solution was added. After an incubation period of 1 h at 37 °C, the staining solution was replaced with fresh prewarmed medium, and the cells were immediately imaged.

Tubulin dye (Abberior Tubulin Live 550) was diluted to a working concentration of 50 nM in DMEM. The culture medium was removed, and a prewarmed staining solution was added together with 1 μM verapamil. After an incubation period of 1 h at 37 °C, the staining solution was replaced with fresh prewarmed imaging medium, and the cells were immediately imaged.

### GUV preparation

GUVs were generated by gel-assisted swelling using polyvinyl alcohol^64^ (PVA). A 5% (wt/wt) PVA solution was prepared in Milli-Q water and heated to 90 °C in a water bath until fully dissolved. To construct an open growth chamber, a rubber O-ring was affixed to a cleaned coverslip using a silicone elastomer (Kwik-Cast, World Precision Instruments). Fifty microliters of the heated 5% PVA solution was coated onto the coverslip and dried completely. Lipid mixtures (DPhPC, 850356, Avanti Research) dissolved in 10 μl of chloroform (1 mg ml^−1^) were deposited onto the dried coverslip and evaporated. Following lipid deposition, the chamber was filled with 15 mM sucrose solution, whose osmolarity was adjusted to match that of the external imaging buffer. GUVs were allowed to form by spontaneous swelling at room temperature. For membrane visualization, 5 μM FM4-64 dye (T13320, Thermo Fisher Scientific) was dissolved in Milli-Q water.

### Statistics and reproducibility

No statistical method was used to predetermine sample size. Sample sizes were determined empirically based on common practice in the field of optical imaging to ensure robust and reliable evaluation of resolution and imaging fidelity. For cell evaluation, at least three independent biological replicates were imaged for each experiment, and we confirmed that all attempts at replication were successful, yielding consistent results. The experiments were not randomized, and investigators were not blinded to group allocation during data collection because the operator was required to actively configure and switch between the proposed and conventional imaging modalities. However, to eliminate subjective bias, quantitative outcome assessments were performed using objective, automated computational algorithms. All quantitative data are presented as mean ± s.d., as indicated in the respective figure legends.

### Reporting summary

Further information on research design is available in the Nature Portfolio Reporting Summary linked to this article.

## Online content

Any methods, additional references, Nature Portfolio reporting summaries, source data, extended data, supplementary information, acknowledgements, peer review information, details of author contributions and competing interests and statements of data and code availability are available at https://doi.org/10.1038/s41592-026-03196-6.

## Supplementary information

Supplementary Information Supplementary Figs. 1–23 and Notes 1–18

Reporting Summary

Supplementary Video 1 Changes in fluorescence image contrast in linearly polarized channels and visualization of the SPIFFI workflow.

Supplementary Video 2 Validation of SPIFFI for imaging mitochondria in a fixed COS-7 cell.

Supplementary Video 3 SPIFFI can resolve 80-nm details when combined with existing fluctuation-based super-resolution imaging techniques.

Supplementary Video 4 SPIFFI can capture rapid cellular dynamics and extract multidimensional information.

## Source data

Source Data Fig. 1 Statistical source data.

Source Data Fig. 2 Statistical source data.

Source Data Fig. 3 Statistical source data.

Source Data Fig. 4 Statistical source data.

Source Data Fig. 5 Statistical source data.

## Data Availability

Demonstration data are available at https://github.com/wg9661/SPIFFI. Source data are provided with this paper.

## Code availability

The SPIFFI code is available at https://github.com/wg9661/SPIFFI.
