# Supplementary material for: SPIFFI enables single-shot super-resolution and multidimensional imaging
Source: Nat Methods. 2026 Aug 31;23(10):2077–86. doi: 10.1038/s41592-026-03196-6 (PMC13645634; doi:10.1038/s41592-026-03196-6)
Supplement: Supplementary file 1 [file 41592_2026_3196_MOESM1_ESM.pdf]

nature methods

Supplementary information

https://doi.org/10.1038/s41592-026-03196-6

SPIFFI enables single-shot super-resolution and multidimensional imaging

In the format provided by the authors and unedited

Contents

1. Microscope configuration 2
2. Imaging model 3
3. Image registration and calibration 5
4 Virtual resampling 8
5 Flowchart of SPIFFI 10
6 PSF size validation using SMLM data 12
7 Fluctuations enhancement in SPIFFI 13
8 Resolution evaluation for different methods 14
9 Simulations 15
10 Potential artefacts and practical considerations 19
11 Fair comparison with other fluctuations-based SR methods 20
12 Fidelity check 20
13 Decorrelation resolution analysis 21
14 Comparison of SPIFFI and SIM 22
15 SPIFFI image of blinking data 23
16 Image drift 24
17 Dipole orientation and DoLP images 25
18 Two-color imaging 26
19 Supplementary References 27

Supplementary Note 1. Microscope configuration

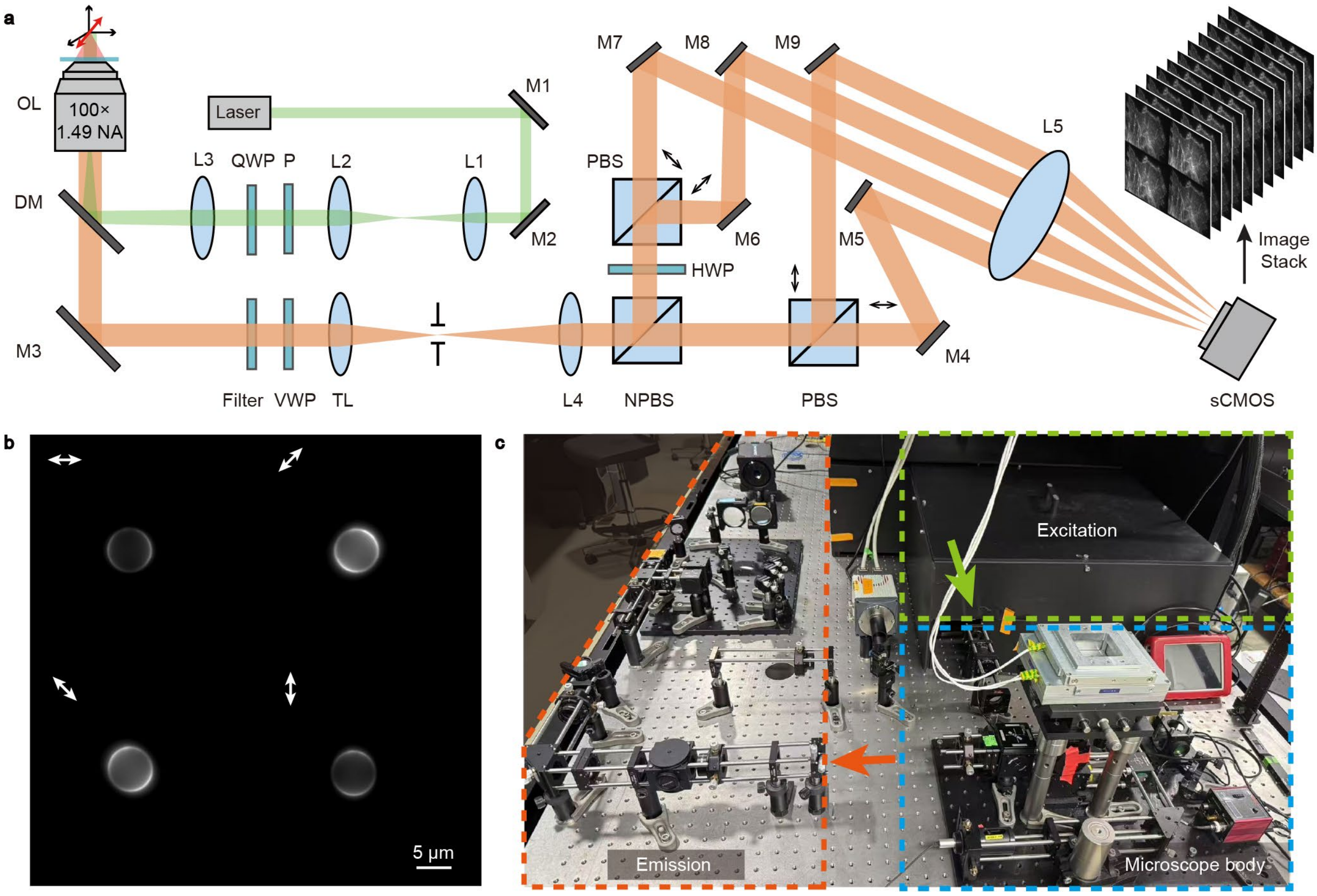

Supplementary Figure 1 Microscope configuration. (a) Schematic diagram. (b) A four-channel image of a GUV as an example on a single camera. (c) Photo of our custom microscope. L1-L4: lens; P: polarizer; QWP: quarter waveplate; M1-M9: mirror; OL: objective lens; DM: dichroic mirror; VWP: variable waveplate; TL: tube lens; NPBS: non-polarizing beamsplitter; PBS1-PBS2: polarizing beamsplitter; HWP: half waveplate. Detailed descriptions can be found in **Methods**.

Supplementary Note 2. Imaging model

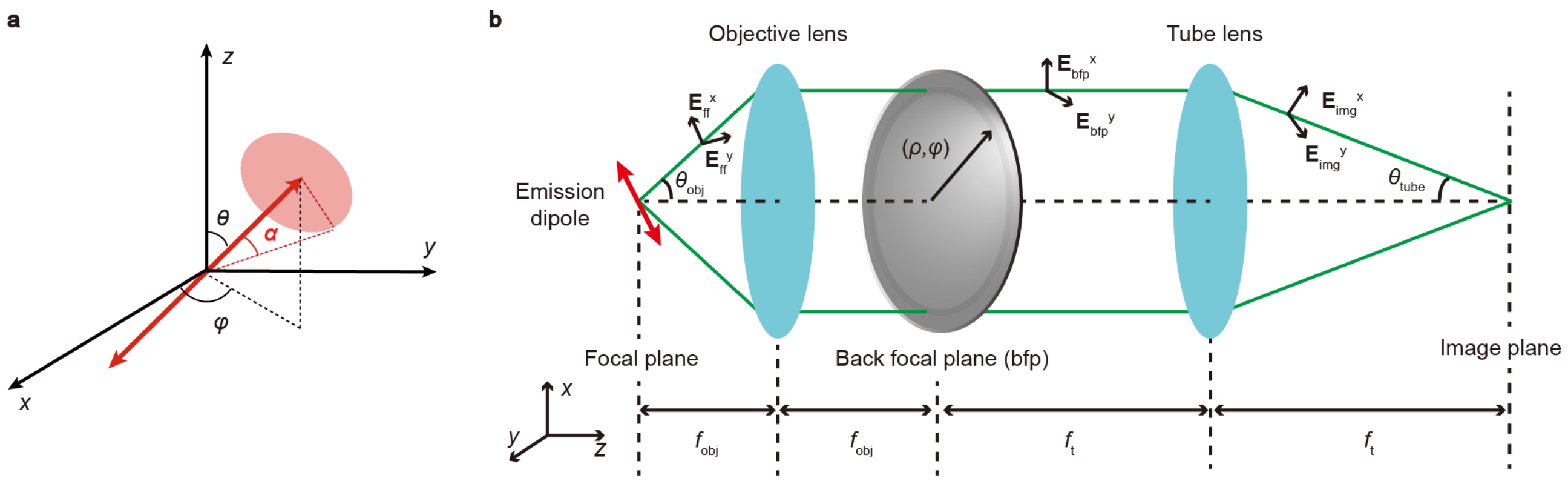

Supplementary Figure 2 Schematic diagram of forward imaging model. (a) Orientation of dipole moment. Azimuth angle φ∈[0°,180°), polar angle θ∈[0°,90°], and wobble angle α∈[0°,90°]. (b) Model of emission path in microscope.

The orientation of fluorescent dipole is defined in spherical coordinates and the corresponding dipole moment is **μ.** The emission path of the microscope is modeled as 4f system and the Green function is used for describing the electric field of fluorescence as shown in **Supplementary Figure 2**. We only summarize the key conclusions here because the basic theory has been modeled in previous references[1-5].

$$\hat{\boldsymbol{\mu}}(\Theta,\Phi)=\begin{bmatrix}\mu_x\\ \mu_y\\ \mu_z\end{bmatrix}=\begin{bmatrix}\sin(\Theta)\cos(\Phi)\\ \sin(\Theta)\sin(\Phi)\\ \cos(\Theta)\end{bmatrix} \qquad \text{Eq 1}$$

$$\mathbf{G}_{\mathrm{ff}}(\theta,\varphi)=\frac{e^{in_1kr}}{4\pi r}\begin{bmatrix}1-\cos^2(\varphi)\sin^2(\theta) & -\cos(\varphi)\sin(\varphi)\sin^2(\theta) & -\cos(\varphi)\cos(\theta)\sin(\theta)\\ -\cos(\varphi)\sin(\varphi)\sin^2(\theta) & 1-\sin^2(\varphi)\sin^2(\theta) & -\sin(\varphi)\cos(\theta)\sin(\theta)\\ -\cos(\varphi)\cos(\theta)\sin(\theta) & -\sin(\varphi)\cos(\theta)\sin(\theta) & 1-\cos^2(\theta)\end{bmatrix} \qquad \text{Eq 2}$$

$$\mathbf{E}_{\mathrm{ff}}(\theta,\varphi,d)=Ae^{in_1kd\cos(\theta)}\mathbf{G}_{\mathrm{ff}}(\theta,\varphi)\hat{\boldsymbol{\mu}}(\Theta,\Phi) \qquad \text{Eq 3}$$

where $\boldsymbol{G}_{ff}$ is Green's formula for the propagation of the electric field ($\mathbf{E}_{ff}$) in the far field, and the parameter *d* in Eq.3 means the defocus distance of the fluorescent dipole. The $\mathbf{E}_{ff}$ is then collected by the objective lens and the $\mathbf{E}_{bfp}$ on the back focal plane is shown in Eq 4, where the $\mathbf{R}_{obj}$ rotating the electric field from a spherical wave to a plane wave.

$$\mathbf{E}_{\mathrm{bfp}}(\theta,\varphi,d)=Ae^{in_1kd\cos(\theta)}\sqrt{\frac{n_1}{n_0\cos(\theta)}}\mathbf{R}_{\mathrm{obj}}(\theta,\varphi)\mathbf{G}_{\mathrm{ff}}(\theta,\varphi)\hat{\boldsymbol{\mu}}(\Theta,\Phi) \qquad \text{Eq 4}$$

Wide-field fluorescence microscopy satisfies the Abbe sine condition, so the propagation of the electric field converged by the tube lens satisfies the paraxial approximation of Fourier optics, and the electric field distribution on the image plane can be written as the following basis function form:

$$\mathbf{I}_{\text{img}} = A^2\left[\mu_x^2 \quad \mu_y^2 \quad \mu_z^2 \quad \mu_x\mu_y \quad \mu_x\mu_z \quad \mu_y\mu_z\right]\begin{bmatrix} |g_x^p|^2 & |g_x^s|^2 \\ |g_y^p|^2 & |g_y^s|^2 \\ |g_z^p|^2 & |g_z^s|^2 \\ 2\mathrm{Re}\left((g_x^p)^* g_y^p\right) & 2\mathrm{Re}\left((g_x^s)^* g_y^s\right) \\ 2\mathrm{Re}\left((g_x^p)^* g_z^p\right) & 2\mathrm{Re}\left((g_x^s)^* g_z^s\right) \\ 2\mathrm{Re}\left((g_y^p)^* g_z^p\right) & 2\mathrm{Re}\left((g_y^s)^* g_z^s\right) \end{bmatrix} \qquad \text{Eq 5}$$

where *p* and *s* represent the parallel and perpendicular electric field components, respectively. The matrix on the right side represents the basis functions of the microscope, and the vector on the left is the second-order moment of the dipole moment. Then, for a four-channel polarimetric system with two sets of orthogonal channels (0°-90°, 45°-135°), we use the Jones matrix to describe the rotation of the polarization state in the second pair of orthogonal channels and write all channels as follows:

$$\begin{aligned}\mathbf{I}_{\text{4channels}} &= A^2\begin{bmatrix} |g_x^0|^2 & |g_y^0|^2 & |g_z^0|^2 & 0 \\ |g_x^{90}|^2 & |g_y^{90}|^2 & |g_z^{90}|^2 & 0 \\ |g_x^{45}|^2 & |g_y^{45}|^2 & |g_z^{45}|^2 & 2\mathrm{Re}\left((g_x^{45})^* g_y^{45}\right) \\ |g_x^{135}|^2 & |g_y^{135}|^2 & |g_z^{135}|^2 & 2\mathrm{Re}\left((g_x^{135})^* g_y^{135}\right) \end{bmatrix}\begin{bmatrix} \mu_x^2 \\ \mu_y^2 \\ \mu_z^2 \\ \mu_x\mu_y \end{bmatrix} \\ &= A^2\mathbf{M}_{\text{m}}(\hat{\boldsymbol{\mu}}\hat{\boldsymbol{\mu}}^{\text{T}})_{\text{sub}}\end{aligned} \qquad \text{Eq 6}$$

where *sub* represents a subset of the second-order moment of the dipole moment. Since the coefficient matrix $\mathbf{M}_{\text{m}}$ of the four-channel system is not full ranked, then the $\mu_x\mu_z$ and $\mu_y\mu_z$ components are not coupled into the system. Finally, considering the rotational mobility of a molecule, when it wobbling uniformly and slowly within a hard cone angle, we can replace the dipole moment $\hat{\boldsymbol{\mu}}$ of the fixed molecule with the wobbling dipole moment $\overline{\mathbf{m}}$, where γ means rotational mobility, which ranges from 0 to 1:

$$\begin{aligned} m_{ii} &= \gamma\mu_i + \frac{1-\gamma}{3} \qquad i = x, y, z \\ m_{ij} &= \gamma\mu_i\mu_j \qquad (i \neq j) \\ \gamma &= 1 - \frac{3\alpha}{2\pi} + \frac{\alpha^2}{2\pi^2} \end{aligned} \qquad \text{Eq 7}$$

Supplementary Note 3. Image registration and calibration

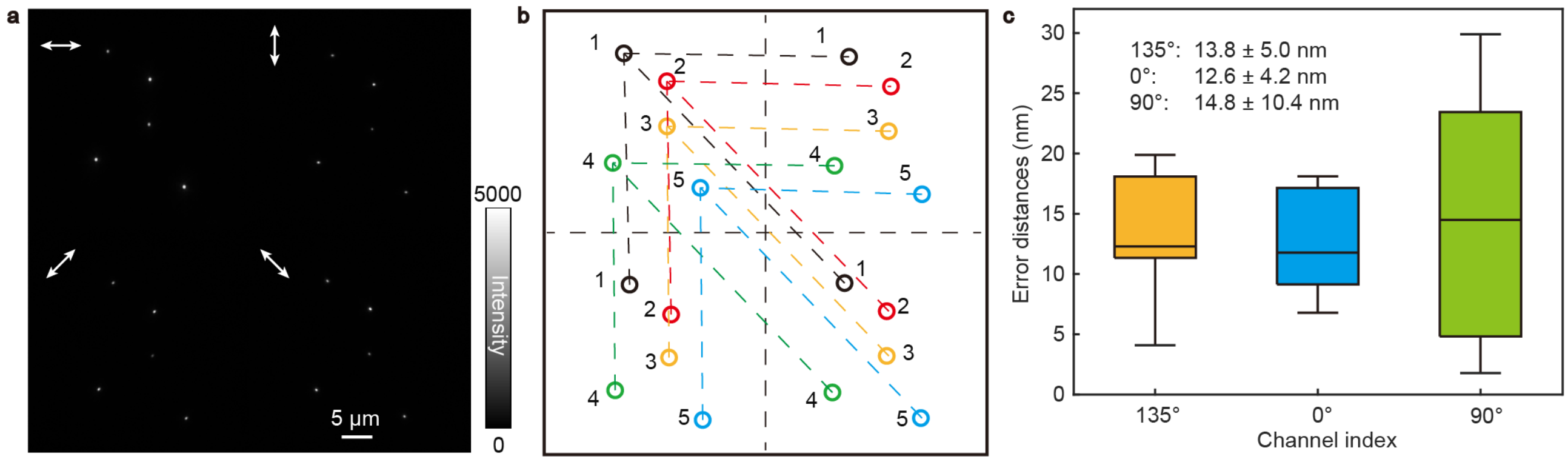

Supplementary Figure 3 Image registration. (a) four-channel images acquired by one camera. (b) PSFs selected for affine transformation registration. (c) distance errors after registration. The statistical result was obtained by analyzing 11 beads evenly distributed in the field of view.

SPIFFI records four polarization channels on a single camera chip (**Supplementary Figure 3a**), requiring geometric registration prior to autocorrelation analysis. We acquired a calibration stack of sparsely distributed fluorescent beads and localized them in every quadrant. Four well-isolated images (**Supplementary Figure 3b**) were used to determine an affine transformation that mapped each polarization channel onto the reference (45°) channel. After transformation, the residual lateral offsets between corresponding centroids were re-evaluated by localization again, yielding registration errors (in Euclidean distance) of 13.8 ± 5.0 nm, 12.6 ± 4.2 nm and 14.8 ± 10.4 nm for the 135°, 0° and 90° channels, respectively (**Supplementary Figure 3c**). These sub-20 nm errors are well below the final SPIFFI resolution limits (≈160 nm for single-shot and ≈80 nm for dual-stage reconstructions), confirming that the observed spatial polarization fluctuations originate from dipole orientation rather than misregistration artifacts.

Then we compensated for polarization distortions introduced by optical coating (e.g. dichroic mirror) through two steps: physical and numerical correction. Physical correction compensates for polarization distortion using variable waveplates and needs to be performed in the optical path, while numerical calibration performs intensity compensation when calculating the dipole angle. When calibrating polarization distortion, we insert a polarizer at the back focal plane in the emission path to generate linearly polarized emitters with a specific in-plane orientation (**Supplementary Figure 4a**). The phase difference caused by the optical coating is compensated by adjusting the retardation of the variable waveplate. Since the direction of the emitter is known, we only need to adjust the intensity of the orthogonal channel to zero. By rotating the direction of the polarizer, we can find an optimal retardation value and maintain this value in subsequent experiments. **Supplementary Figure 4b** shows images of a horizontally oriented emitter before and after polarization compensation, with significant differences in fluorescence intensity and PSF size in the 45° detection

channel (**Supplementary Figure 4c**). Important to note is that polarization compensation is performed within the optical path, so this aspect does not need to be considered in SPIFFI reconstruction.

Numerical correction involved calibrating the relative fluorescence intensities of the four detection channels using fluorescent beads. For this step, an additional polarizer was temporarily inserted at the back focal plane to generate a virtual and perfectly linearly polarized PSF. By rotating the polarizer to four orientations (0°, 90°, 45°, 135°), we normalized the gain of each analyzer channel. Since the dipole azimuthal angle φ is calculated from the Stokes vector of polarized light (Eq 8 & Eq 9), only two sets of orthogonal detection channels needed to be calibrated independently (Eq 10), where e and t represent the experimental and theoretical values, respectively. The superscript denotes the polarizer angle, and the subscript indicates the analyzer channel. However, the impact of the imbalance on SPIFFI is usually small compared to polarization distortion (**Supplementary Figure 4d**). We can solve this problem using the pre-deconvolution, and improve the accuracy of orientation retrieval by compensating during the calculation of dipole angles

$$\begin{cases} S_1 = (I_0 - I_{90})/(I_0 + I_{90}) \\ S_2 = (I_{45} - I_{135})/(I_{45} + I_{135}) \end{cases} \quad \text{Eq 8}$$

$$\begin{cases} \varphi = 0.5 \times \tan^{-1}(S_2/S_1) \\ DoLP = \sqrt{(S_1)^2 + (S_2)^2} \end{cases} \quad \text{Eq 9}$$

$$\begin{cases} \begin{bmatrix} I_{e0}^{0} & I_{e0}^{90} \\ I_{e90}^{0} & I_{e90}^{90} \end{bmatrix} = \mathbf{M}_{\mathrm{PSA}(0-90)} \begin{bmatrix} I_{t0}^{0} & I_{t0}^{90} \\ I_{t90}^{0} & I_{t90}^{90} \end{bmatrix} \\ \begin{bmatrix} I_{e45}^{45} & I_{e45}^{135} \\ I_{e90}^{135} & I_{e90}^{135} \end{bmatrix} = \mathbf{M}_{\mathrm{PSA}(45-135)} \begin{bmatrix} I_{t45}^{45} & I_{t45}^{135} \\ I_{t90}^{135} & I_{t90}^{135} \end{bmatrix} \end{cases} \quad \text{Eq 10}$$

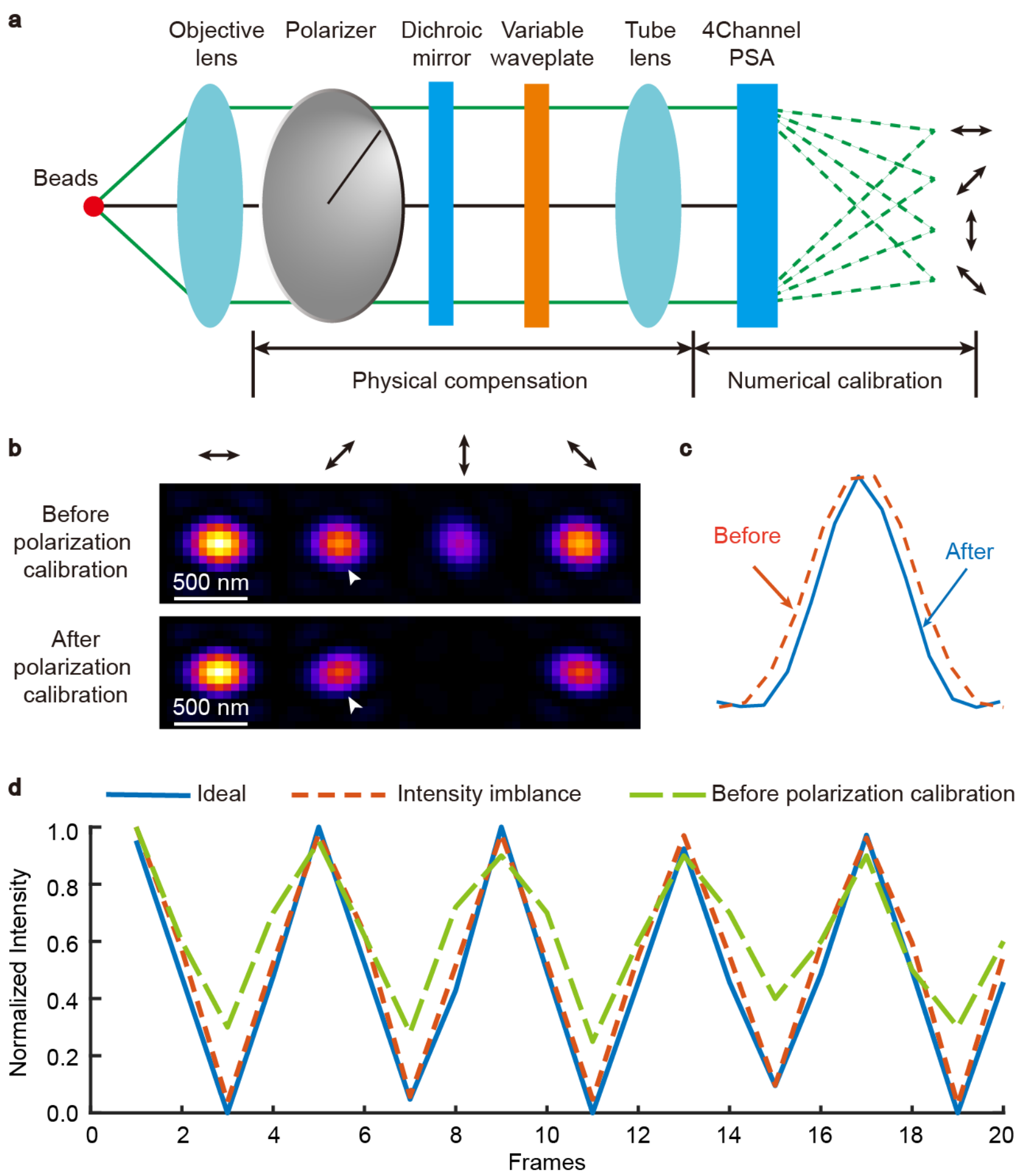

Supplementary Figure 4 Polarization distortion calibration in the emission path. (a) Calibration diagram, generating an ideal in-plane linearly polarized dipole by inserting a polarizer at the back focal plane. PSA: polarization state analyzer. (b) Demonstration of a horizontally oriented emitter before and after calibration. Polarization distortion affects not only the intensity in each channel but also the size of the polarized PSF. (c) Comparison of PSF diameters in the 45° channel; the difference in size is approximately 30 nm. (d) Comparison of the intensity fluctuations in the center of the PSF across different channels: ideal case (blue), intensity ratio imbalance caused by PSA (red), and polarization distortion caused by the coating on the dichroic mirror (green).

Supplementary Note 4. Virtual resampling

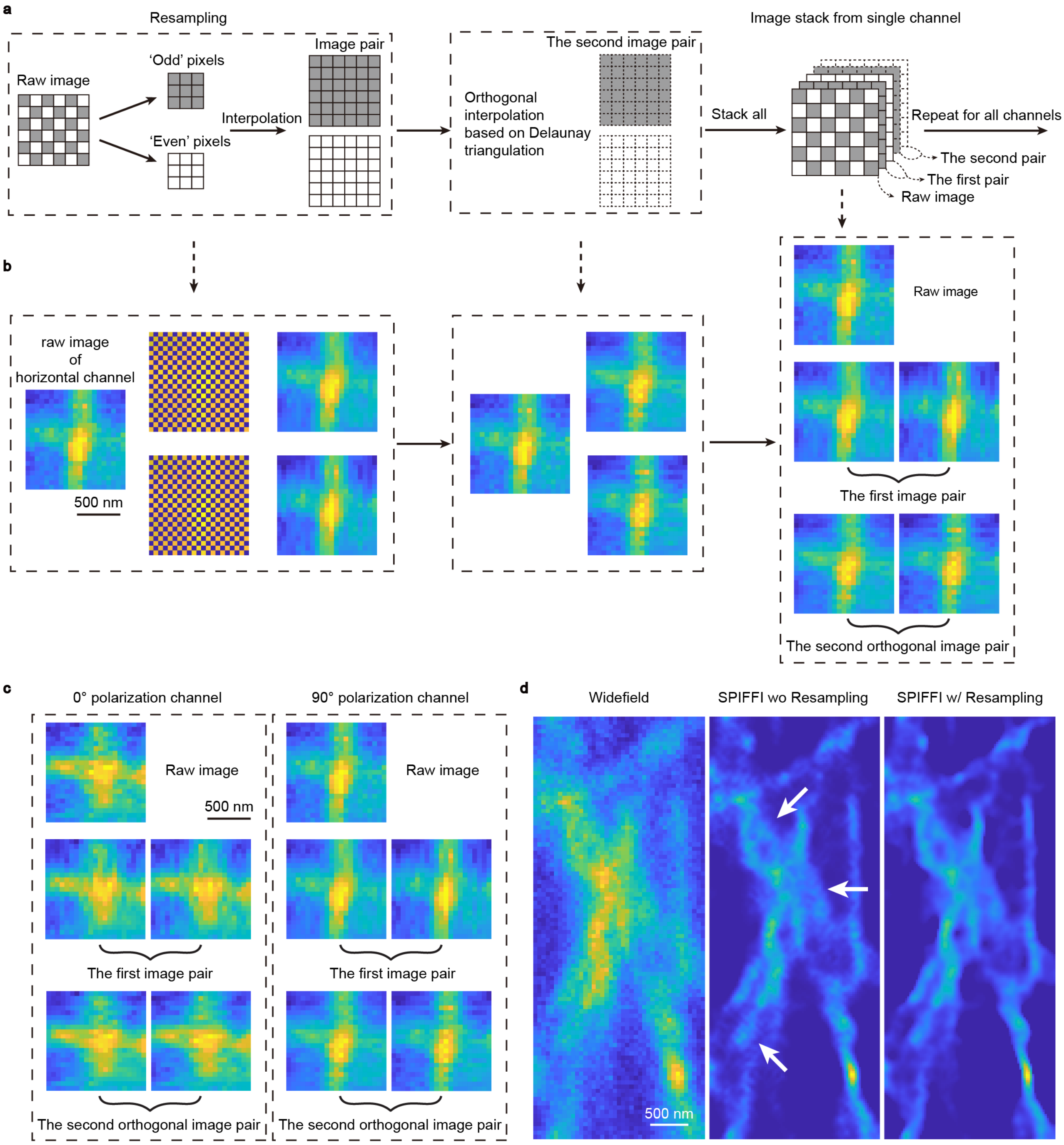

Supplementary Figure 5 Virtual resampling. (a) detailed description of virtual resampling. The raw image is downsampled in a checkerboard pattern and then interpolated back to its original size. Each downsampled image undergoes Delaunay triangulation-based interpolation in two orthogonal directions. (b) take the fluorescence image of microtubules as an example. (c) comparison of images from 0° and 90° polarization channels. (d) comparison of SPIFFI with and without the resampling step. Resampling significantly reduces image artifacts and maintains structural continuity.

In order to maintain the continuity of the target structure and reduce artifacts caused by image noise, we introduced a virtual resampling step before correlation calculation. The procedure operated on each channel independently and consisted of three steps (**Supplementary Figure 5a & b**):

1. **Checkerboard down-sampling:** The original image *I(x,y)* was divided into two interleaved sub-lattices: even pixels (2*m*, 2*n*) and odd pixels (2*m*+1, 2*n*+1), creating two statistically independent noise-paired images.
2. **Pixel interpolation:** Each sub-image was up-sampled back to the original grid using Delaunay triangulation-based interpolation. This interpolation preserves the contrast of local edges and align with the shape of Polar-PSF, thus generating two virtual resampled images. However, since the Delaunay triangulation method is direction-sensitive, our checkerboard-like sampling allows for two orthogonal directions to fill the blank areas in each sub-image. Therefore, we employ the third step: "Orthogonal directions enhancement" to fully leverage fluctuations in light intensity.
3. **Orthogonal directions enhancement for receptive-field augmentation:** To enlarge the receptive field used in subsequent correlation, the same down-sample/interpolate routine was repeat on the orthogonal direction of same image. Combining the original and resampling pairs yielded four virtual replicas per channel.

Supplementary Figure 5c shows a patch of microtubules labelled with CF568. Left and right panels display the horizontal (0°) and vertical (90°) analyzer channels, respectively, which shows polarized images of the same structure with different resolutions and contrasts. Without virtual resampling, SPIFFI image (**Supplementary Figure 5d**) produces a honeycomb pattern and edge discontinuities along the microtubules. After virtual resampling the edges were smoothed and the artefact was suppressed. *It's important to note that resampling only reduces artifacts in the reconstruction but does not increase image resolution.*

Supplementary Note 5. Flowchart of SPIFFI

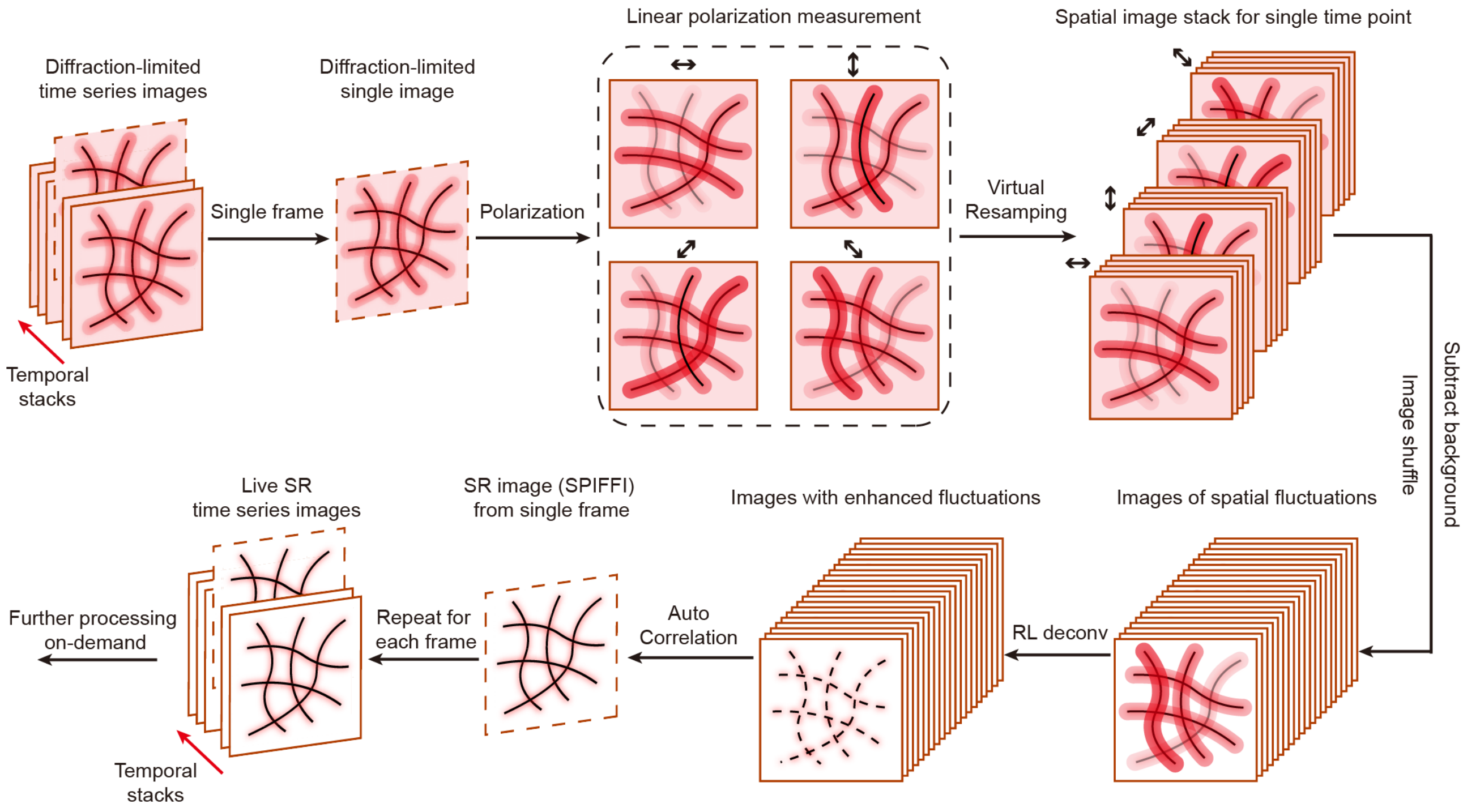

Supplementary Figure 6 the workflow of SPIFFI. The workflow begins with a single widefield exposure split into four polarization-resolved channels. Virtual resampling, background subtraction and image shuffle are applied to each channel to generate noise-paired images for data augmentation. A stack of spatially intensity fluctuating images are then processed through deconvolution and autocorrelation to yield the final super-resolved image. Each single-shot super-resolution image reconstructed by SPIFFI can be used for further time temporal fluctuation analysis for further resolution improvement.

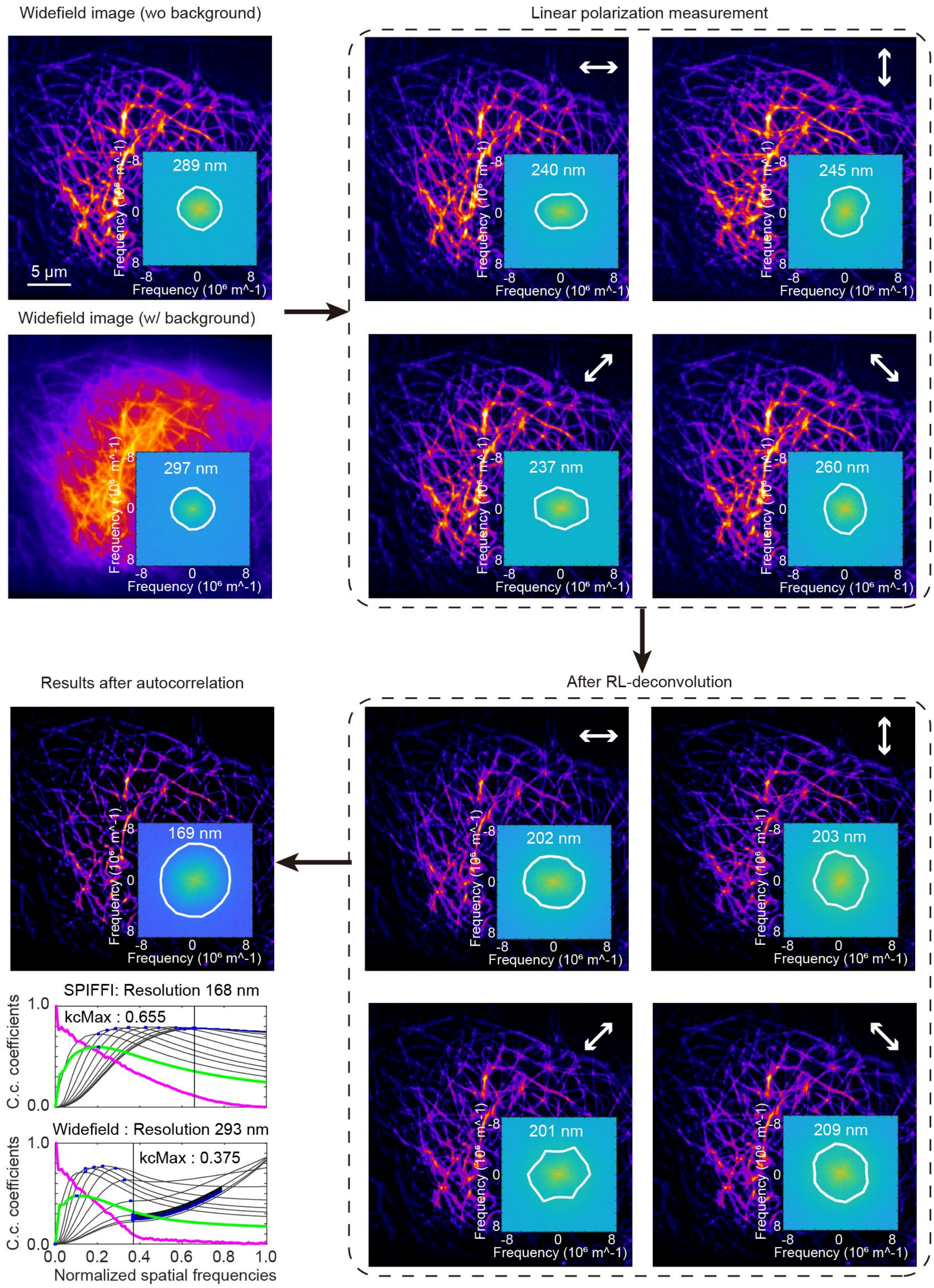

Supplementary Figure 7 Detailed resolution contributions of individual steps in SPIFFI reconstruction. The resolution boundaries on the Fourier spectrum are calculated by the sectorial decorrelation analyze, and the corresponding resolution value is obtained by taking the average of the top three maximum frequencies from 9 directions. The sectorial resolution of SPIFFI is similar to the global decorrelation result of the whole image (bottom left corner). Background subtraction contributes little to the resolution improvement, but polarization measurement directly improves the resolution by approximately 40 nm relative to wide-field images. The 2D Fourier transform image reveals that the resolution improvement in polarization measurement exhibits anisotropy (elliptical boundary). But the results of SPIFFI reconstruction exhibit good isotropy (circular boundary). Therefore, the resolution results obtained from directional analysis and global analysis are similar.

Supplementary Note 6. PSF size validation using SMLM data

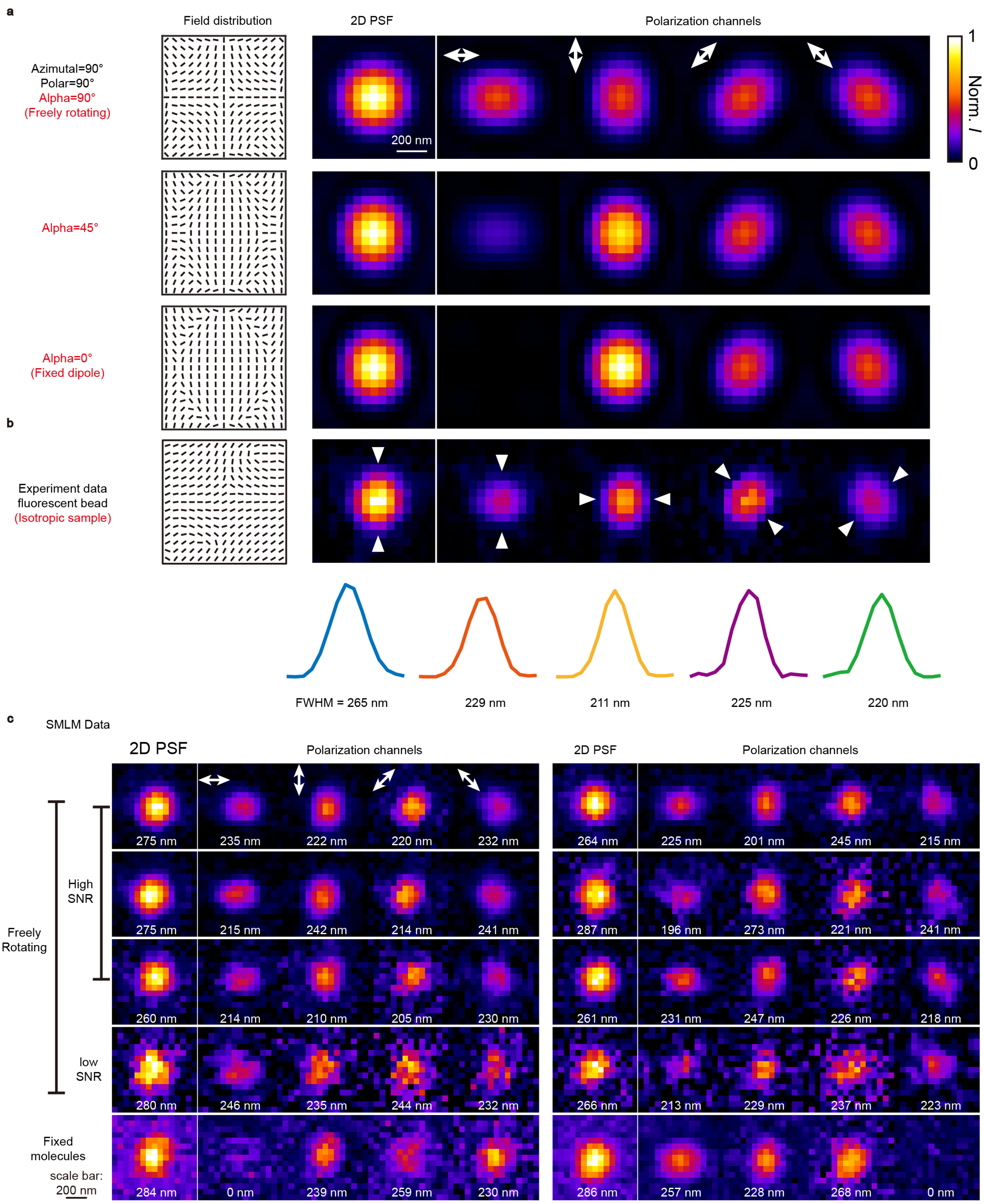

Supplementary Figure 8 (a) Simulation of dipoles with different wobbling angles. Parameter α represents the half cone angle when molecules are considered wobbling within hard cone regions. The short bars in the first column represents the polarization state distribution of the PSF within the corresponding pixels. (b) Experimental data of a 100 nm fluorescent bead. (c) SMLM data indicates that the reduction in PSF size also works for freely rotating molecules, with the waist beam size of each PSF shown at the bottom

Supplementary Note 7. Fluctuations enhancement in SPIFFI

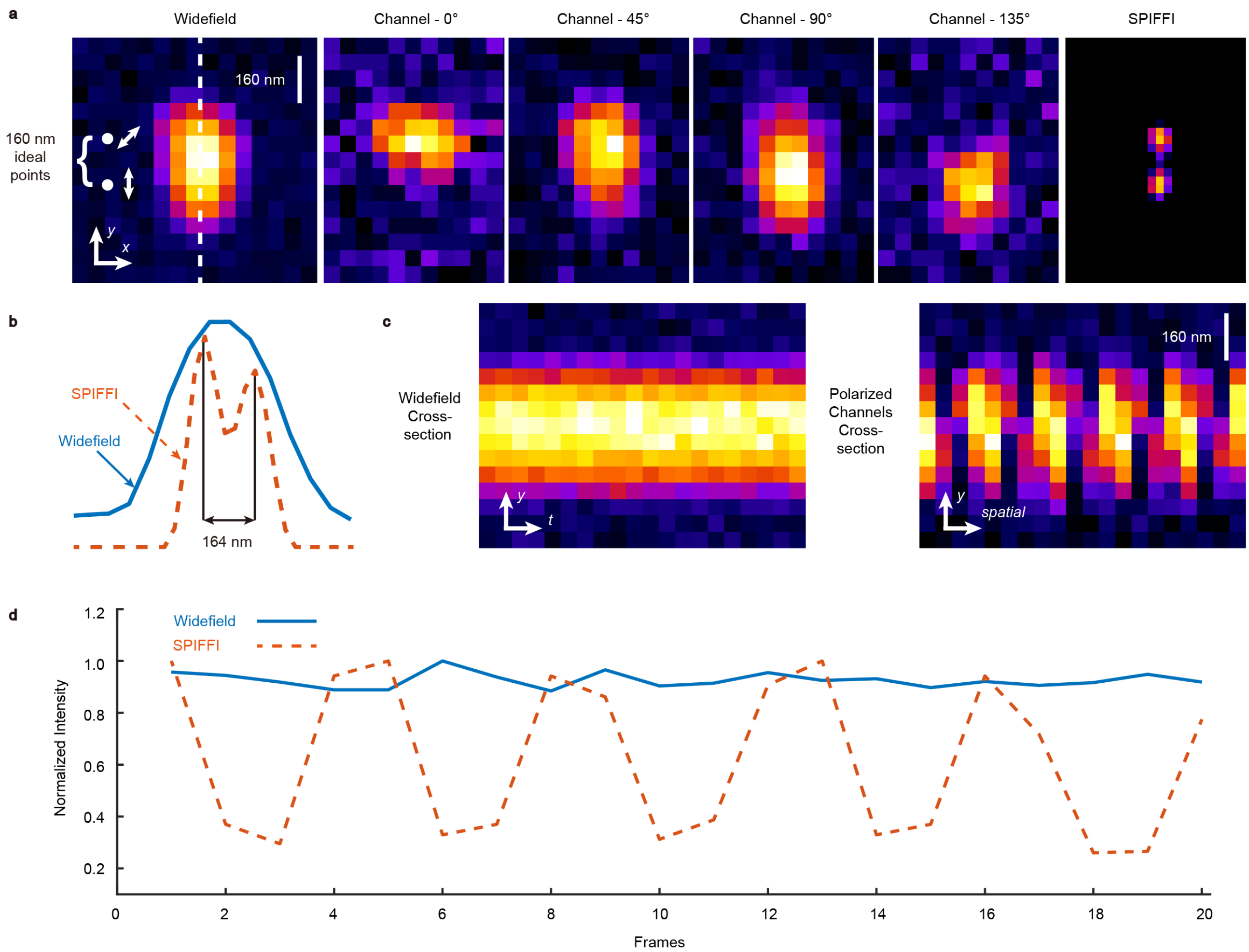

Supplementary Figure 9 Simulation of resolution enhancement mechanism in SPIFFI. (a) Simulated emission of two molecules separated by 160 nm with different dipole orientations (indicated by arrows) under 600 nm emission wavelength and 1.49 NA objective lens. While indistinguishable in the pseudo-widefield image, the two emitters show distinct spatial intensity distributions and PSF size across the four polarization channels due to their unique Polar-PSFs. (b) Normalized intensity profiles along the dashed line in (a). SPIFFI (dashed orange) successfully resolves the two peaks at 160 nm, whereas the widefield (solid blue) does not. (c) Spatiotemporal cross-sections along the dashed line comparing the temporal fluctuations of widefield signals versus the spatial fluctuations of polarized signals introduced in 20 frames. The intensity fluctuations in 20 widefield images were simulated by adding random noise, while the polarized fluctuations originate from resampling across four channels. (d) Intensity fluctuations at the midpoint between two molecules in 20 frames of images. This confirms that the combination of polarization contrast and virtual resampling creates the high-contrast fluctuations required for resolution enhancement through autocorrelation

Supplementary Note 8. Resolution evaluation

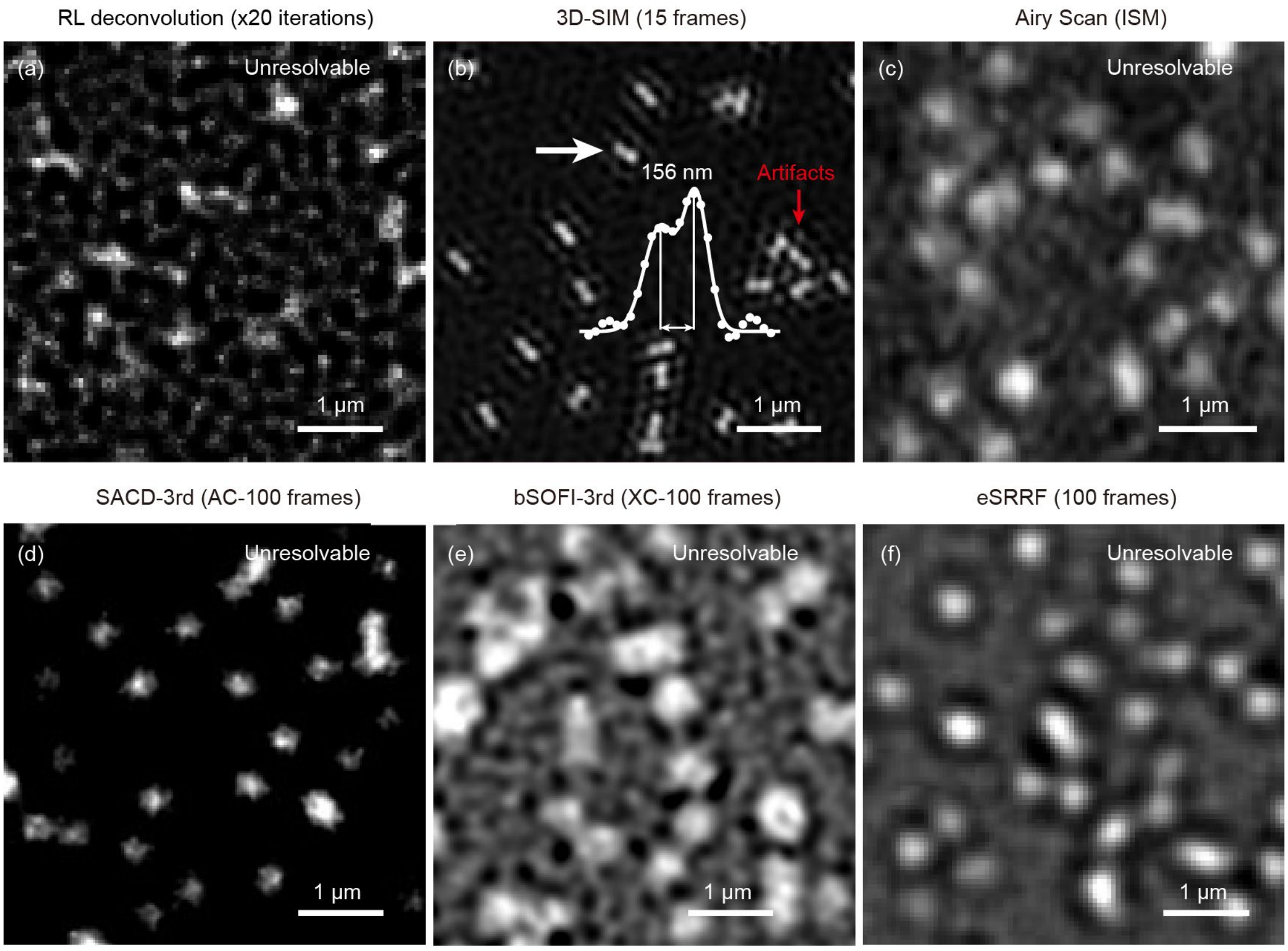

Supplementary Figure 10 Resolution evaluation for different super-resolution methods using 160 nm nanorulers. (a) Richardson–Lucy deconvolution stopped at 20 iterations. (b) 3D-SIM with 15 frames for reconstruction. (c) Airy-Scan microscope (commercial version of ISM). (d) SACD with 100 frames. (e) bSOFI with 100 frames. (f) eSRRF with 100 frames.

Supplementary Note 9. Simulations

In this section we evaluated the resolution of SPIFFI by simulating double lines that varied in line spacing, dipole state, and noise level. Simulation parameters were as follows: The emission wavelength was 600 nm, the numerical aperture of the objective lens was 1.49, the dipole spacing along each line was 20 nm, pixel size in the image plane was 40 nm and all dipoles emitted simultaneously within a single frame. Because both dipole orientation and polarized PSFs needed to be modeled, WF images for each polarization channel were generated by placing individual dipoles at prescribed positions, rather than convolving an ideal line with isotropic 2D Gaussian functions.

In addition, we preset the pixel size in image plane to 10 nm during simulation and then down-sampled[6] to 40 nm, which ensured that both dipoles spacing and line spacing conformed to Nyquist's sampling theorem and avoided positional shifts of straight lines caused by direct coarse-pixel simulation. Finally, the noise model included two parts: Poisson noise and Gaussian noise, and overall noise level was quantified by the peak signal-to-noise ratio (PSNR) of the WF image. WF images shown in the simulations were obtained by summing four polarization channels.

Ideal, noise-free results (**Supplementary Figure 11**):

SPIFFI resolved two parallel dipole lines down to ~140 nm and the limit improved slightly to ~120 nm for perpendicular dipoles, but the large wobble angle did not significantly degrade the resolution. Fluorescence-intensity variations caused by dipole orientation were visible in the PSF images in each panel, and red markers were used to indicate resolution levels.

Noise robustness (**Supplementary Figure 12**):

With increasing noise, resolution degraded only modestly (~20 nm) for the lines with stationary dipoles and SPIFFI maintained 160–180 nm resolution even at PSNR = 15. To fully utilize the temporal continuity of fluorescent images, a three-frame sliding window could smooths cusp artifacts and preserves structural fidelity, though it does not improve resolution.

Wobbling dipoles (**Supplementary Figure 13**):

The performance of SPIFFI did not drop significantly for high PSNR images (PSNR=25) with large wobble angles. However, compared with **Supplementary Figure 12**, it can be seen that the stationary dipole has a 20-40 nm resolution improvement over the large-angle wobbling dipole. Therefore, rigidly linked fluorescent probes were expected to be more robust to noise than long-chain labeling methods.

Finally, SPIFFI in this simulation relied solely on spatial fluctuations, without incorporating temporal intensity variations. This avoided issue associated with nonlinear temporal fluorescence fluctuations

and made the method more efficient than traditional multi-frame techniques. It was important to note that the goal here is to evaluate the resolution performance of SPIFFI; therefore, although simulating smaller line spacings could yield more 'accurate' results, such adjustments were not necessary for this validation.

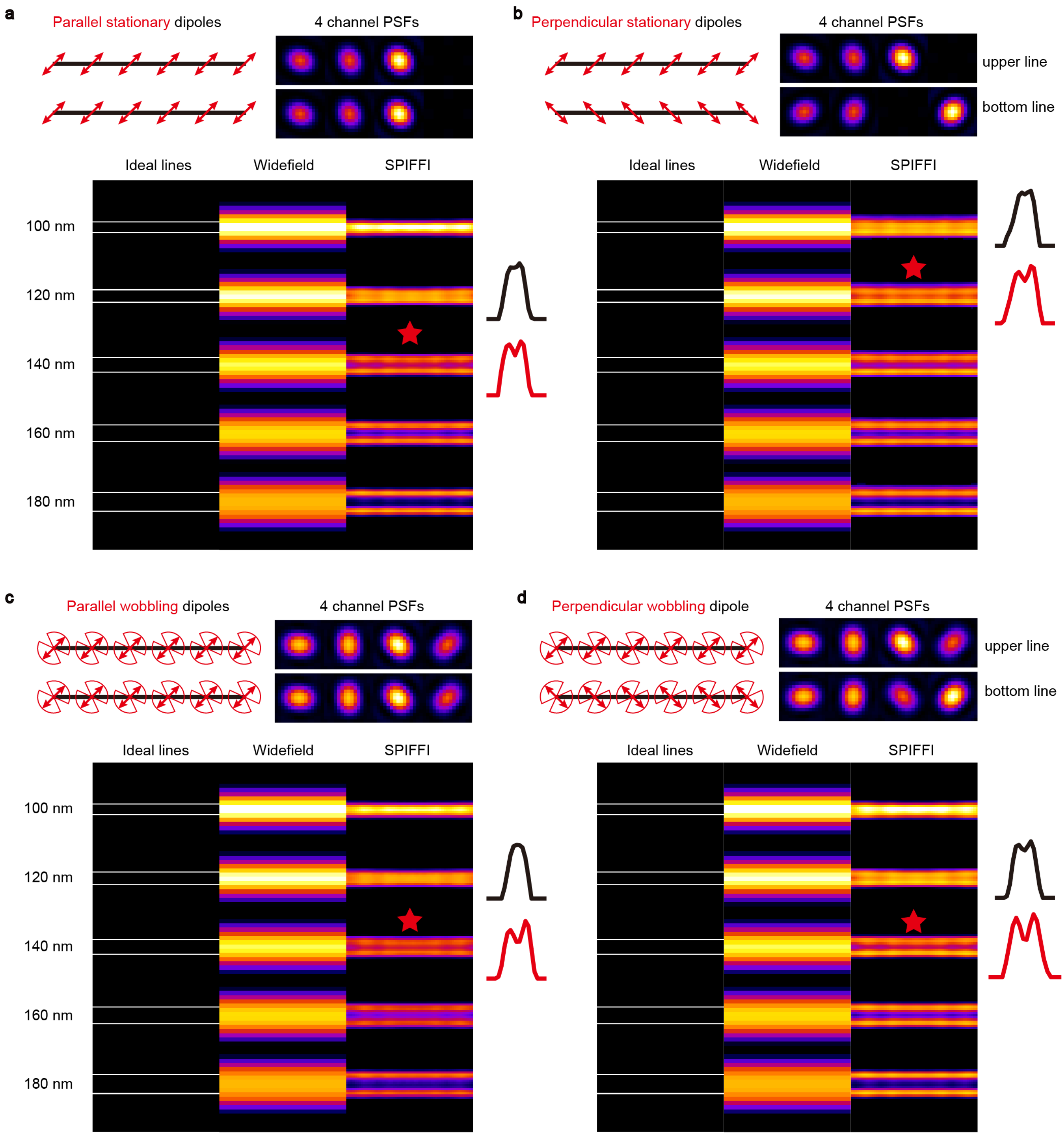

Supplementary Figure 11 Double-line simulations with different dipole orientation. Red intensity profiles and stars represent the minimum resolvable distance, and the black profiles represent the maximum unresolvable distance. (a) Schematic diagram of two straight lines with parallel and stationary dipoles, four-channel polarized PSFs and SPIFFI results. (b) Double-line simulation with perpendicular and stationary

dipoles. (c) Double-line simulation with parallel and wobbling dipoles (wobbling angle α=70°). (d) Double-line simulation with perpendicular and wobbling dipoles (wobbling angle α=70°).

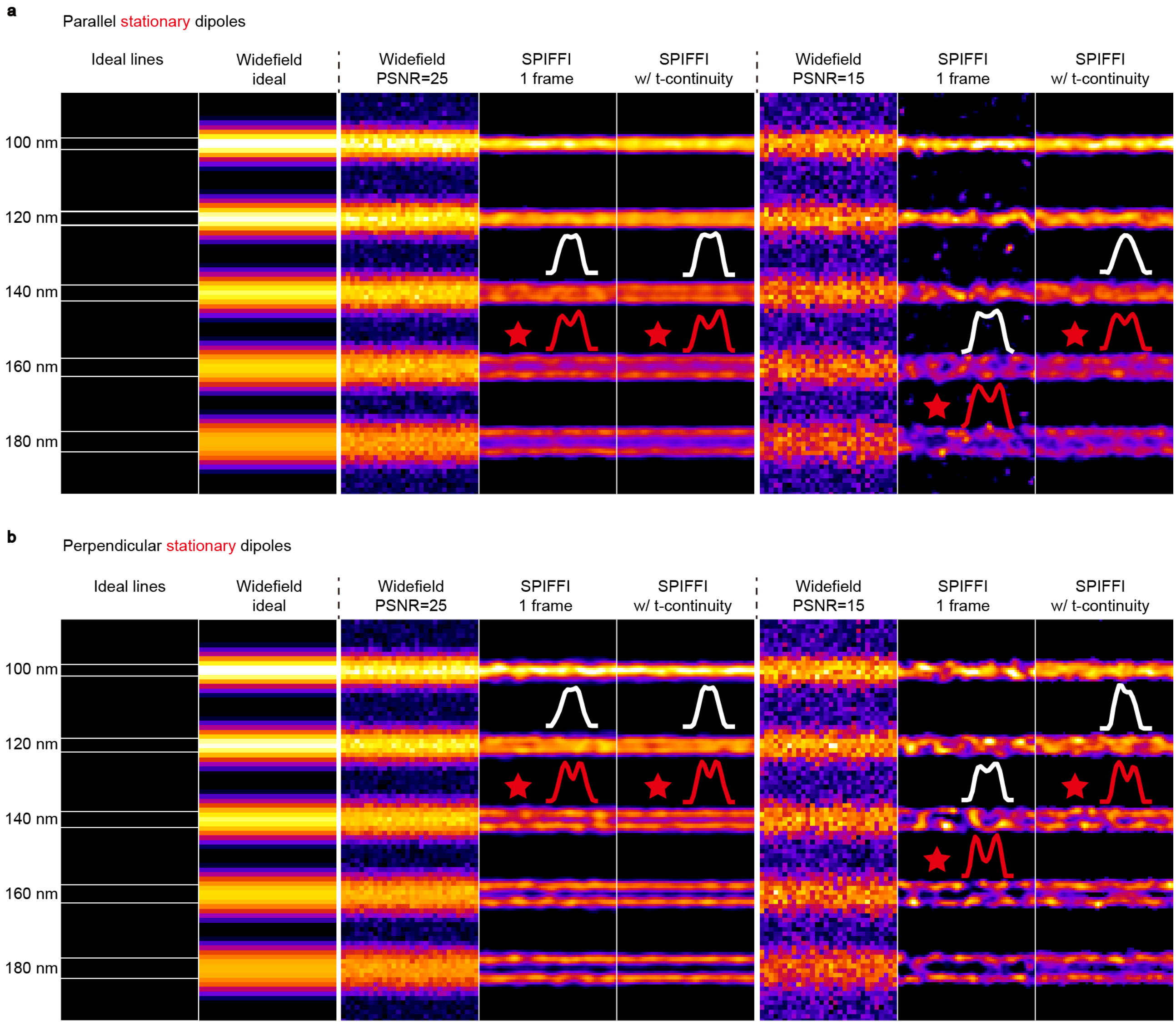

Supplementary Figure 12 Simulation of two stationary dipole lines under different noise levels. Red intensity profiles and stars represent the minimum resolvable distance, and white profiles represent the maximum unresolvable distance. (a) Simulation of parallel dipoles at PSNR=15 and 25. (b) Simulation of perpendicular dipoles at PSNR=15 and 25.

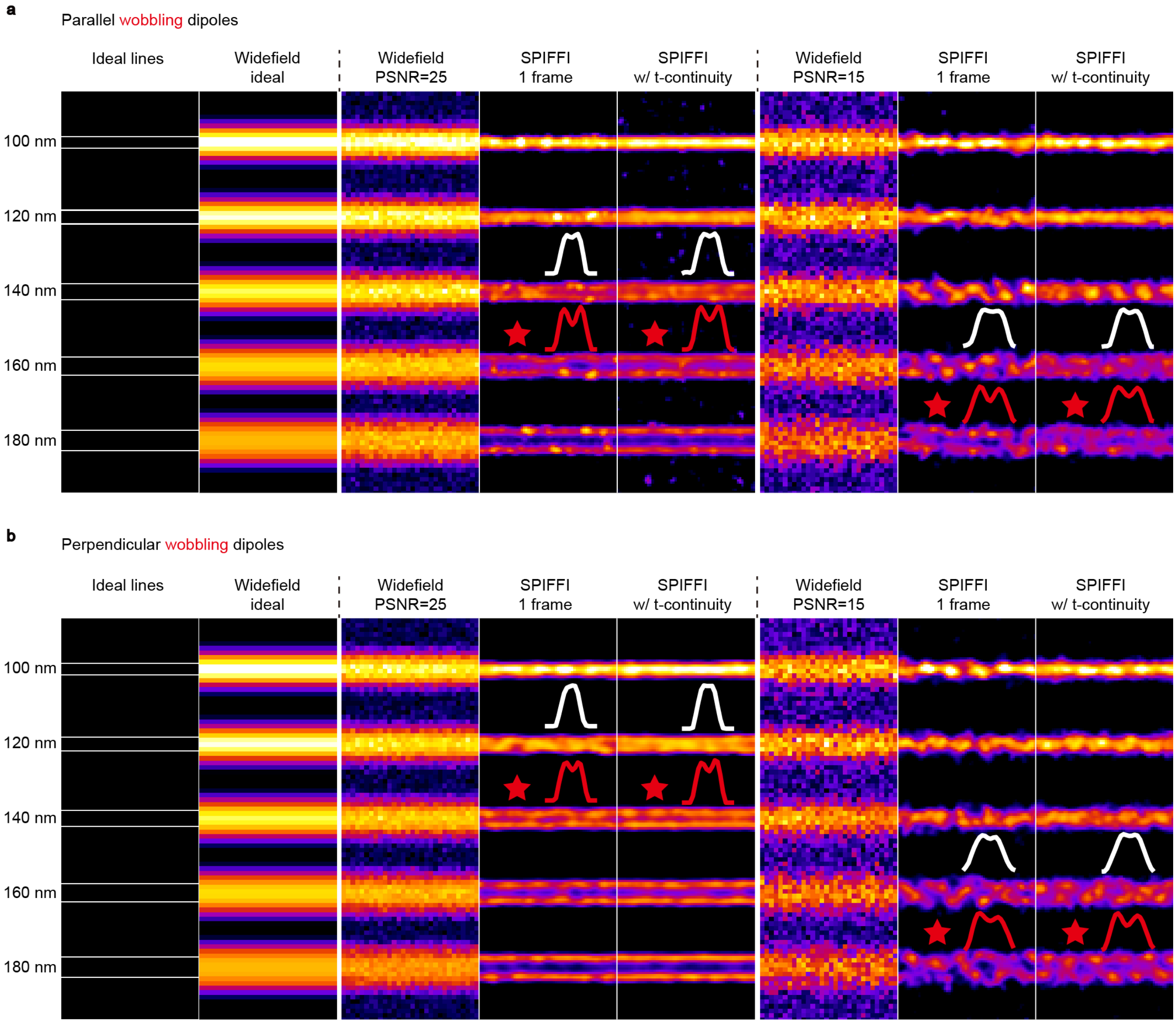

Supplementary Figure 13 Simulation of two wobbling dipole lines under different noise levels. Red intensity profiles and stars represent the minimum resolvable distance, and white profiles represent the maximum unresolvable distance. (a) Simulation of parallel dipoles at PSNR=15 and 25. (b) Simulation of perpendicular dipoles at PSNR=15 and 25.

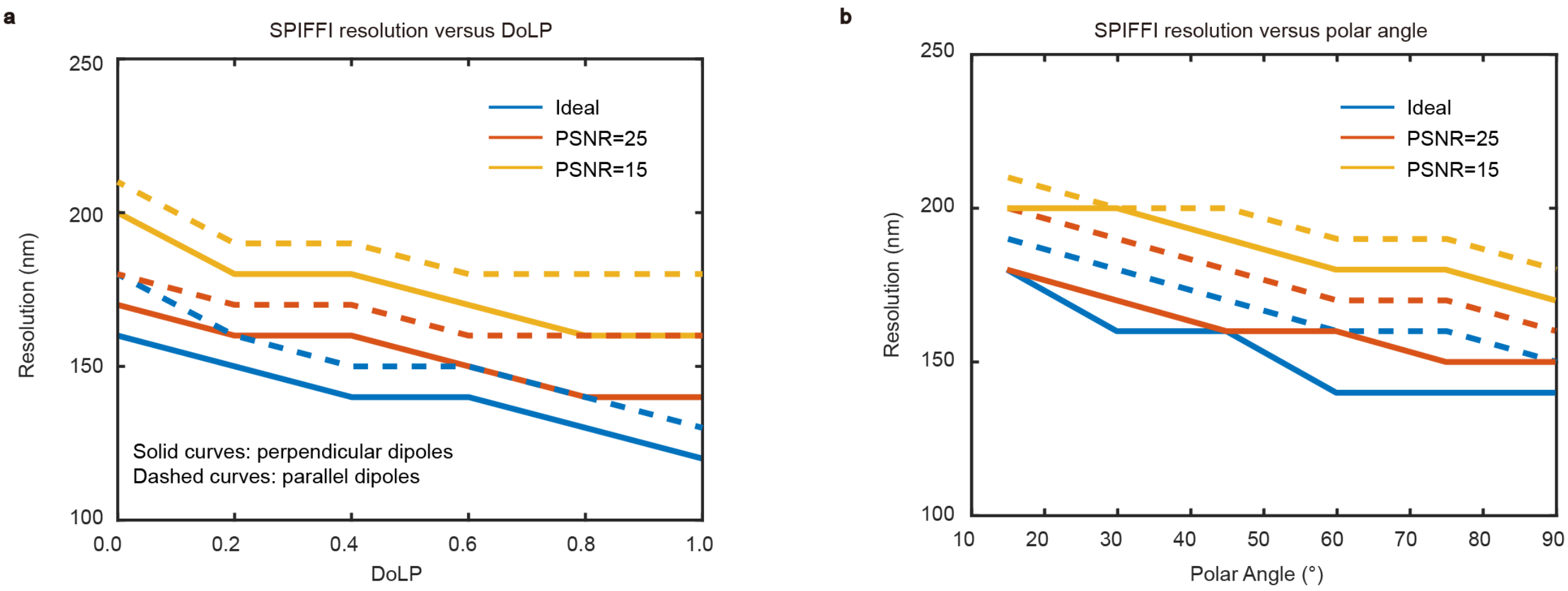

Supplementary Figure 14 (a) Impact of DoLP on resolution (b) Impact of off-plane angle on resolution. The dipole is parallel to the focal plane when the polar angle is 90°, and the wobble angle in the simulation is 70°

Supplementary Note 10. Potential artefacts and practical considerations

In SPIFFI reconstruction, the quality of reconstruction and the achievable resolution are sensitive to several sources. We expanded the discussion by categorizing them into two groups: general factors, which common to fluctuation-based imaging, and SPIFFI-specific factors.

(1) General factors affecting fluctuation-based imaging

Pixel Size: To capture the subtle fluctuations, the effective pixel size at the sample plane must satisfy the Nyquist criterion not only for the diffraction-limited PSF and ideally for the super-resolved PSF. Insufficient sampling leads to spatial aliasing, which obscures high-frequency information required for reconstruction.

Exposure time and photon statistics: Exposure time determines the photon counts collected per frame and therefore the SNR of the recorded fluctuations. Since fluctuation-based methods rely on estimating higher-order statistics or modulation amplitudes, sufficient photon statistics are required for stable reconstruction. Short exposure times may result in photon-limited noise, while excessively long exposures may average out the relevant temporal fluctuations.

(2) SPIFFI-specific factors

Polarization distortion in the optical system: Unexpected retardance in the polarization optics can distort the measured polarization signals, affecting the accurate retrieval of molecular orientations.

Rotational mobility of fluorophores: The rotational dynamics of fluorophores influence the polarization-dependent fluctuation contrast. Highly mobile emitters (large wobble angles) reduce polarization anisotropy (low DoLP) and therefore decrease the fluctuation contrast. But, both the simulation and the isotropic nanorulers in our work show that SPIFFI can achieve a 160 nm resolution even in low DoLP cases.

Supplementary Note 11. Fair comparison with other fluctuations-based SR methods

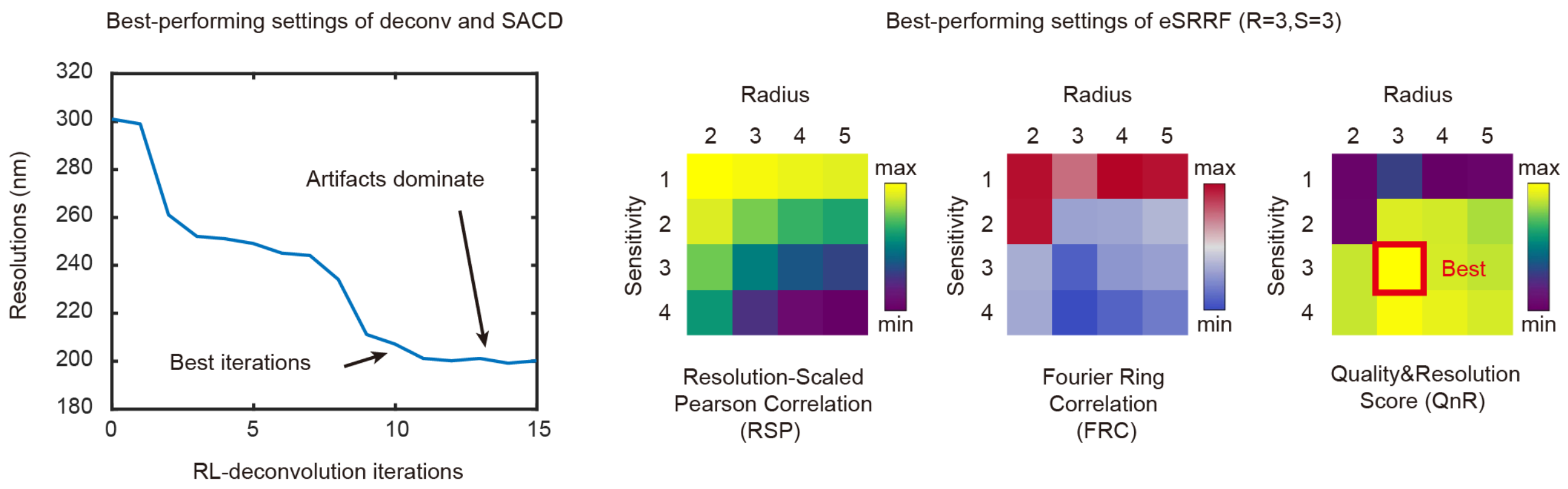

Supplementary Figure 15 Best reconstruction parameters used in comparison. RL-deconv, SACD, and SPIFFI reconstruction all use the same number of deconvolutions to ensure fair comparison. eSRRF guarantees optimal performance by selecting the best QnR parameters.

Supplementary Note 12. Fidelity check

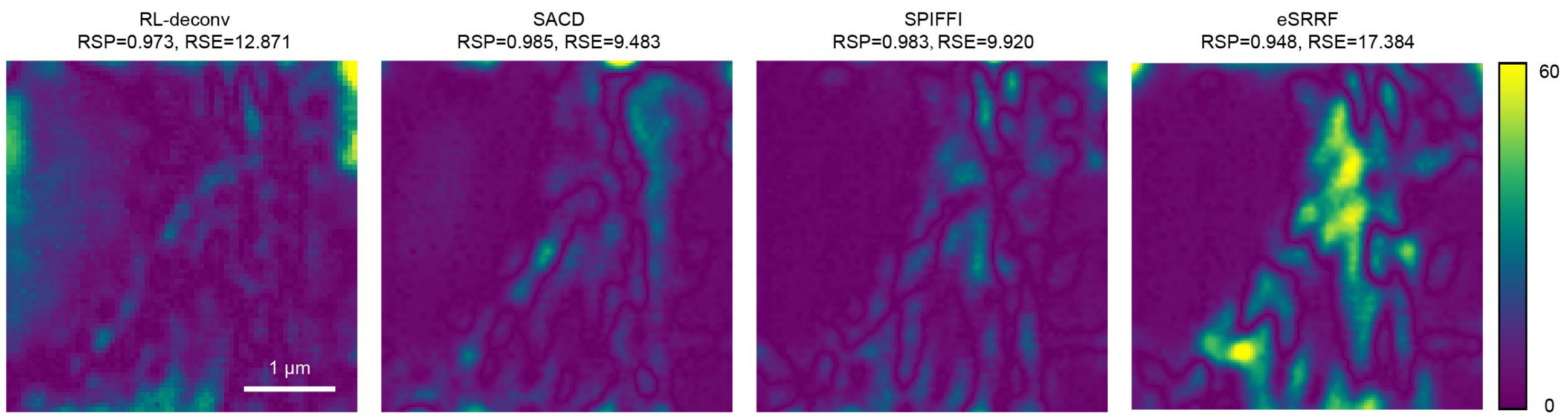

Supplementary Figure 16 Resolution-Scaled Error maps evaluated with SQUIRREL[7]. All images are displayed in the same color scale. RSP: resolution-scaled Pearson coefficient; RSE: resolution-scaled error. High RSP and low RSE represent a good reconstruction.

Supplementary Note 13. Decorrelation resolution analysis

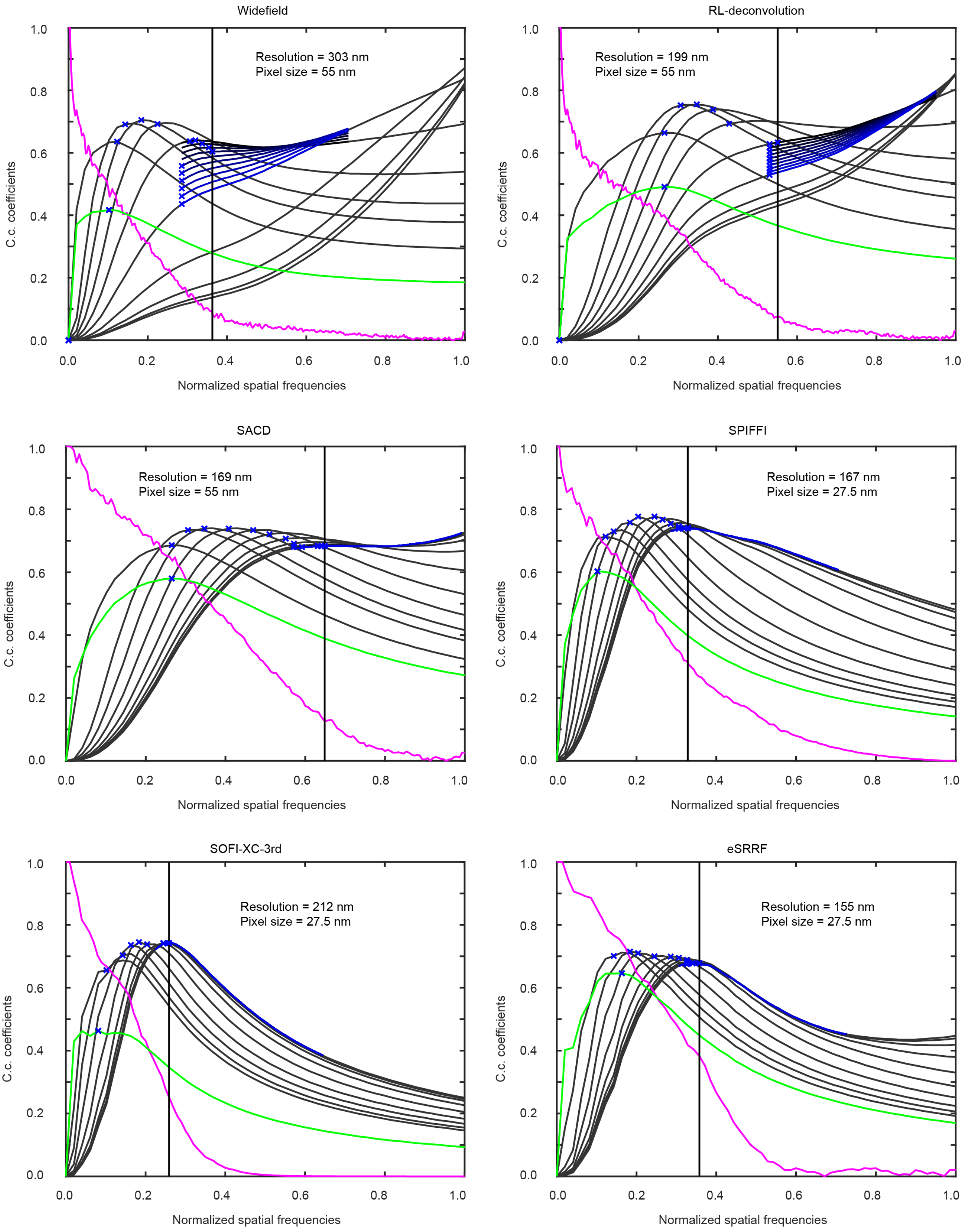

Supplementary Figure 17 Decorrelation resolution analysis of fluorescence images. The single frame resolutions are: 303nm (Widefield), 199 nm (RL-deconv), 167nm (SPIFFI), 169nm (SACD), 212nm (SOFI-XC) and 155 nm (eSRRF) respectively.

Supplementary Note 14. Comparison of SPIFFI and SIM

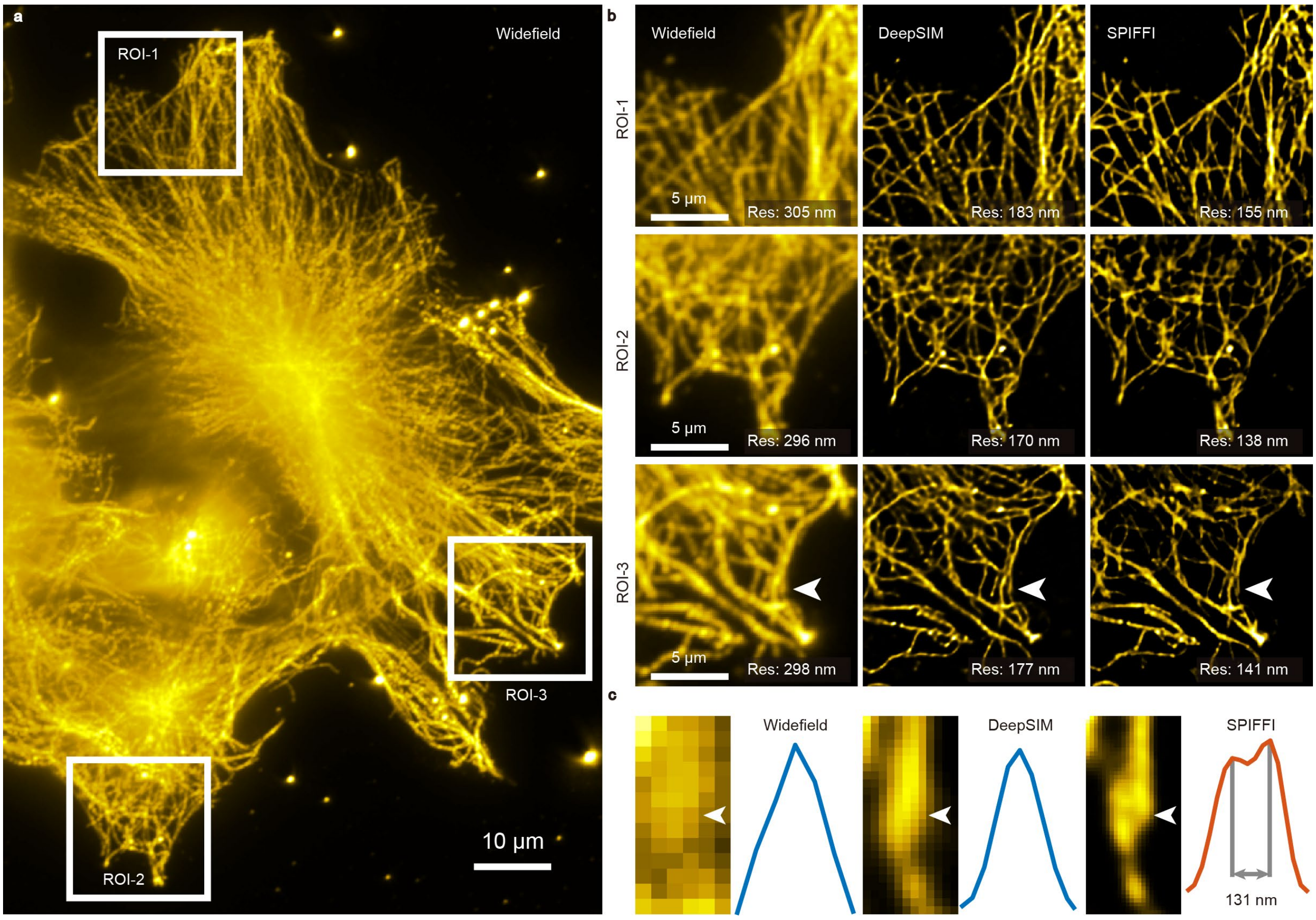

Supplementary Figure 18 Comparison of SPIFFI and SIM performance on immunofluorescence-labeled microtubules. Cos-7 cells were cultured on gold-marked coverslips and mounted with a mounting medium (Fluoromount-G) to enable multi-instrument imaging. SIM data was acquired using the Nikon CrestOptics DeepSIM and was performed by scanning five layers from the bottom with a step of 100 nm, and then the most similar layer was demonstrated. Affine transformation was applied on SPIFFI and SIM to ensure both are displayed at the same scale (a) Widefield image of immunofluorescence labeled microtubules; (b) Super-resolution image comparison of selected ROIs. Each region is repeated ten times independently. (c) two filaments with a spacing of 131 nm were resolved by SPIFFI in ROI-3.

Supplementary Note 15. SPIFFI image of blinking data

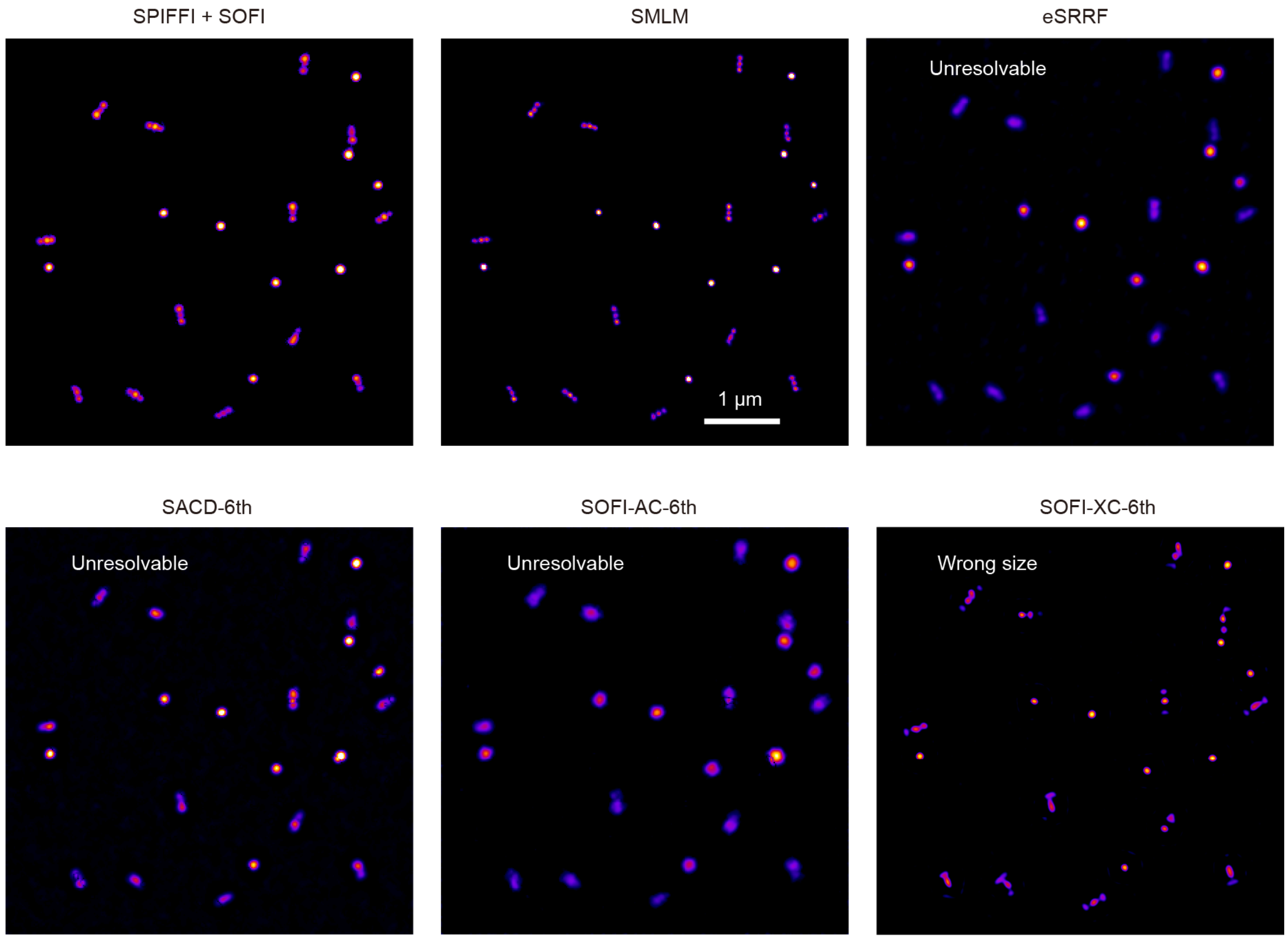

Supplementary Figure 19 The fluctuation-based super-resolution technology was tested on 80 nm nanorulers. All fluctuation-based reconstructions were calculated using 1000 frames. eSRRF, SACD, SOFI-AC and SOFI-XC are not able to correctly resolve the structure of the sample.

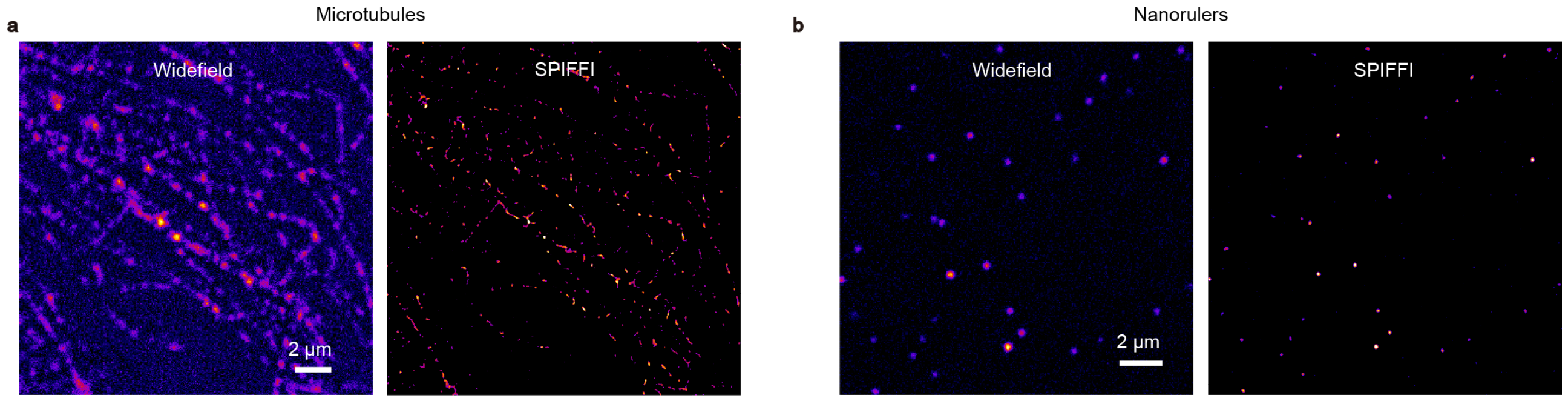

Supplementary Figure 20 Single frame SPIFFI results for the microtubules and DNA-PAINT based 80 nm blinking nanorulers.

Supplementary Note 16. Image drift

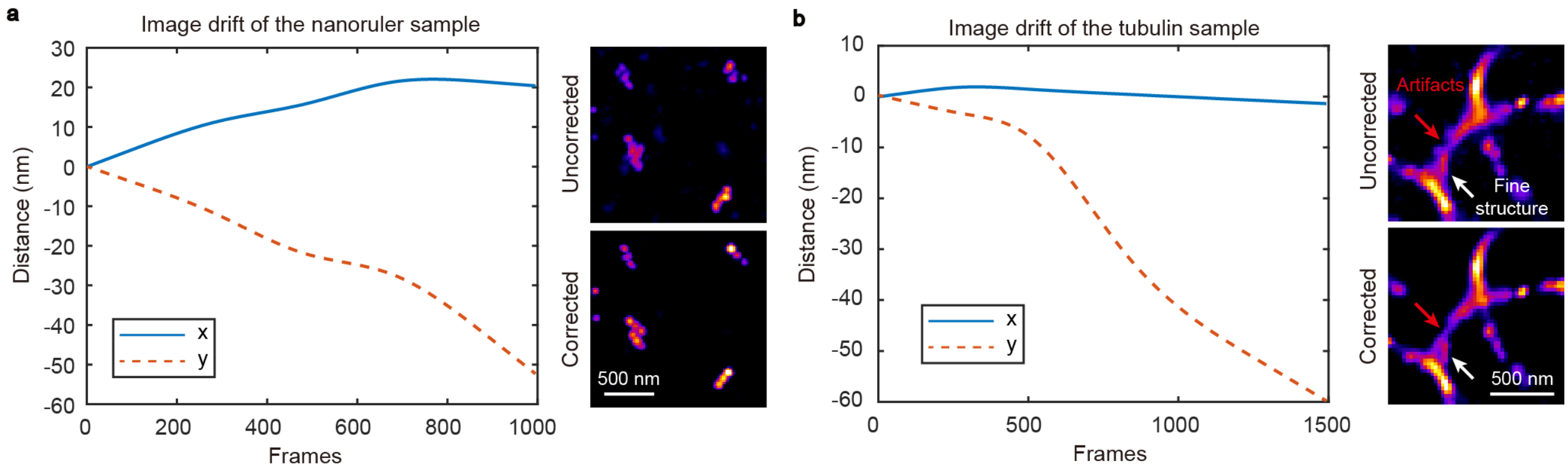

Supplementary Figure 21 The impact of image drift on super-resolution reconstruction. (a) Image drift on 80 nm nanorulers. (b) Image drift on microtubules. Red arrow marks artifacts caused by image drift and white arrow indicates fine structures resolved after drift calibration.

Supplementary Note 17. Dipole orientation and DoLP images

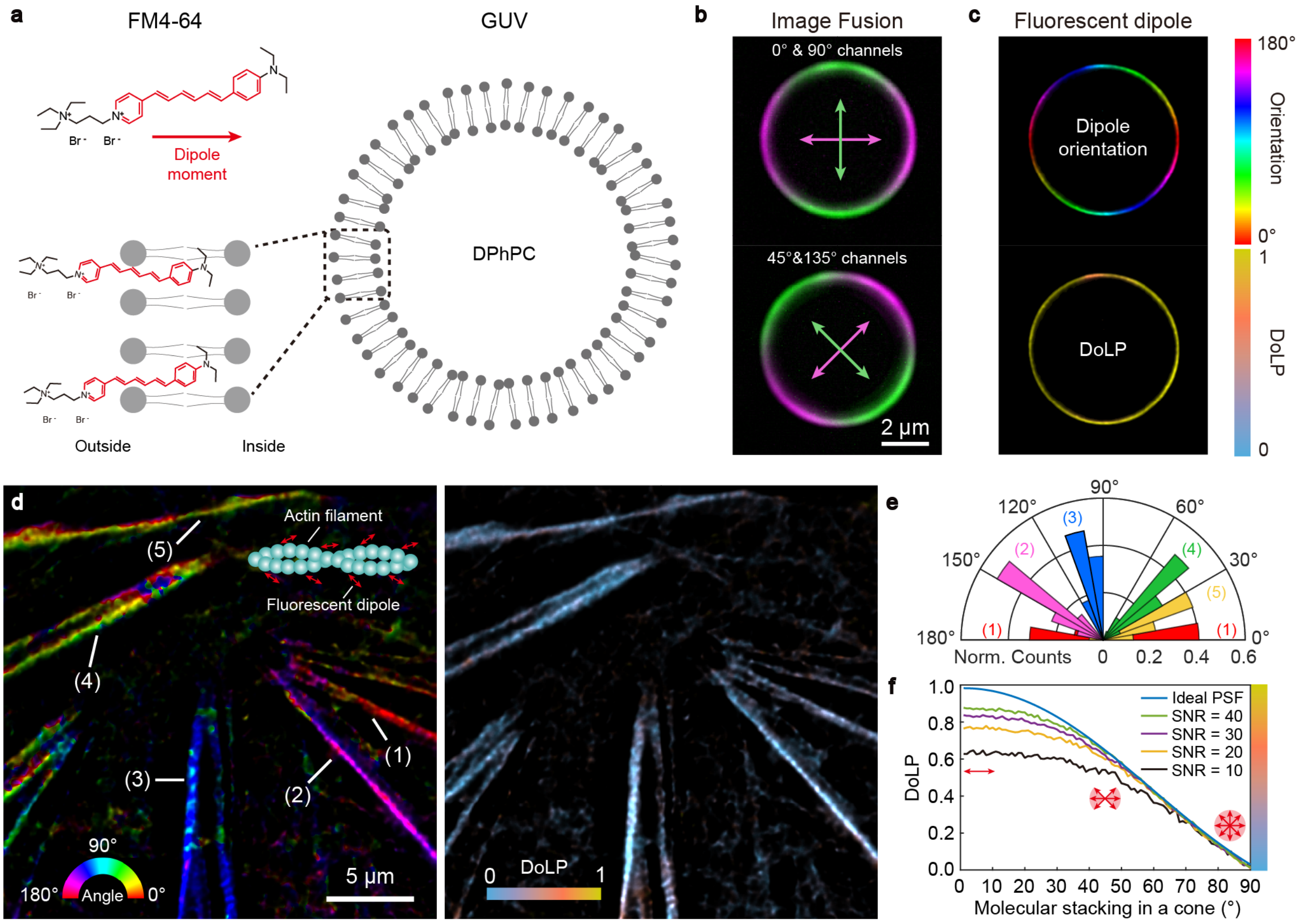

Supplementary Figure 22 Dipole orientation and DoLP images of GUV and actin. (a) Illustration of a GUV model and configuration of fluorescent molecules at the equatorial plane. (b) Fusion images of orthogonal polarization channels, where arrows represent polarization detection directions. (c) Fluorescence dipole orientation and DoLP images at the equatorial plane. FM4-64 molecules are perpendicular to the membrane on average and the depolarization in the DoLP image shows that dyes are not completely fixed to the surface. (d) Fluorescence dipole orientation and DoLP images of COS-7 actins. The orientation of the fluorescent dipoles is similar to that of actin filaments, but the fiber bundles cause severe molecular stacking and thus weaken the polarization degree. (e) Dipole orientations of five representative region on actin filaments. (f) The DoLP decreases due to molecular stacking, where the average orientation of the molecules is assumed to be uniformly distributed within a hard cone. Each sample was tested three times independently.

Supplementary Note 18. Two-color imaging

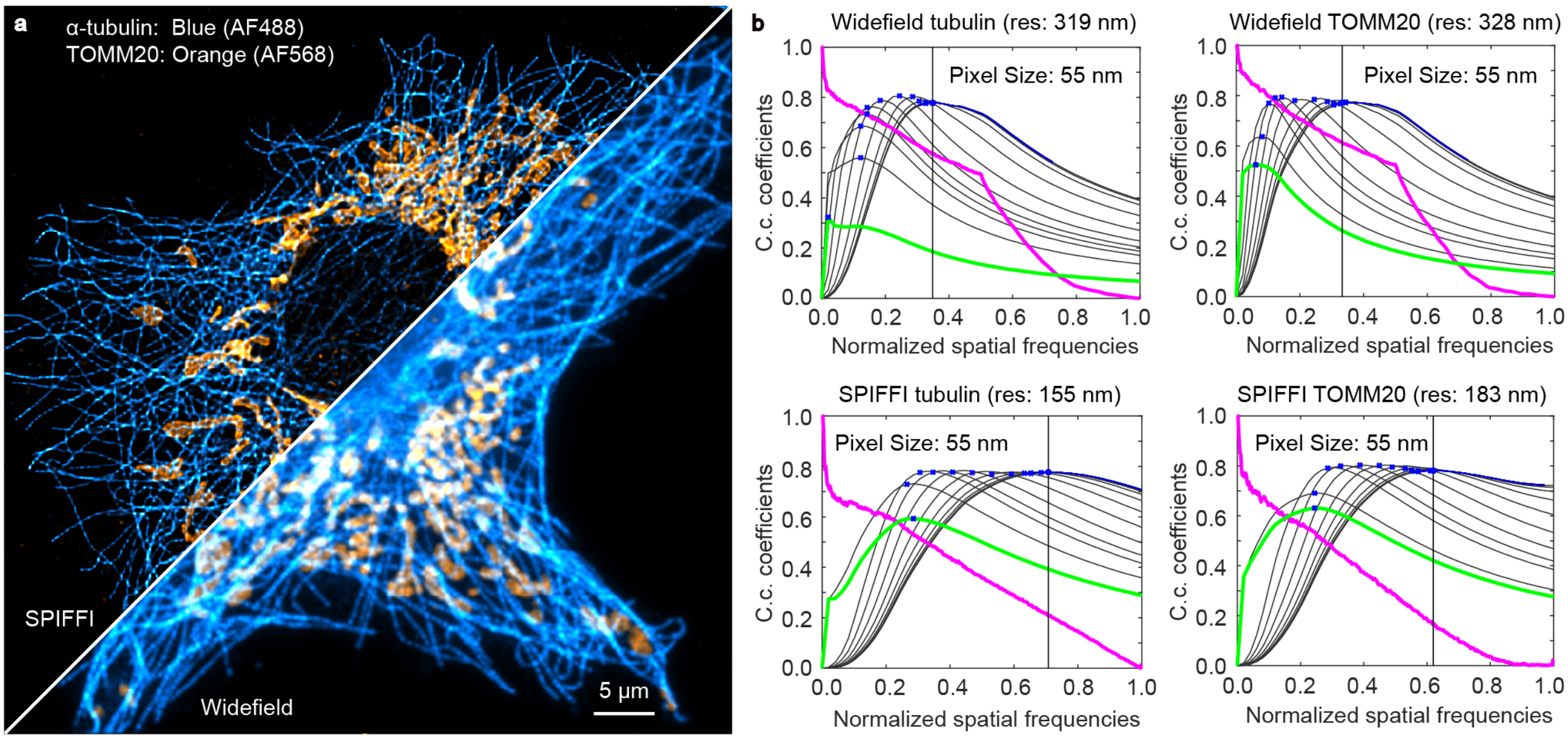

Supplementary Figure 23 Two-color imaging of a cos-7 cell. (a) Comparison of wide-field and SPIFFI images. (b) Decorrelation analysis of images of microtubules (α-tubulin) and mitochondria (TOMM20). The resolution of microtubules improved from 319 nm to 155 nm, and the resolution of the mitochondrial outer membrane improved from 328 nm to 183 nm.

Supplementary References

1 Backer, A. S. & Moerner, W. E. Extending Single-Molecule Microscopy Using Optical Fourier Processing. *The Journal of Physical Chemistry B* **118**, 8313-8329 (2014). https://doi.org/10.1021/jp501778z

2 Backer, A. S. & Moerner, W. E. Determining the rotational mobility of a single molecule from a single image: a numerical study. *Optics Express* **23**, 4255-4276 (2015). https://doi.org/10.1364/OE.23.004255

3 Zhang, O., Lu, J., Ding, T. & Lew, M. D. Imaging the three-dimensional orientation and rotational mobility of fluorescent emitters using the Tri-spot point spread function. *Applied Physics Letters* **113** (2018). https://doi.org/10.1063/1.5031759

4 Guo, W. *et al.* Dipole orientation reveals single-molecule interactions and dynamics on 2D crystals. *arXiv preprint arXiv:2408.01207* (2024).

5 Rimoli, C. V., Valades-Cruz, C. A., Curcio, V., Mavrakis, M. & Brasselet, S. 4polar-STORM polarized super-resolution imaging of actin filament organization in cells. *Nature Communications* **13**, 301 (2022). https://doi.org/10.1038/s41467-022-27966-w

6 Sage, D. *et al.* Super-resolution fight club: assessment of 2D and 3D single-molecule localization microscopy software. *Nat. Methods* **16**, 387-395 (2019). https://doi.org/10.1038/s41592-019-0364-4

7 Culley, S. *et al.* Quantitative mapping and minimization of super-resolution optical imaging artifacts. *Nat. Methods* **15**, 263-266 (2018). https://doi.org/10.1038/nmeth.4605
